# The functional impact of rare variation across the regulatory cascade

**DOI:** 10.1016/j.xgen.2023.100401

**Published:** 2023-09-06

**Authors:** Taibo Li, Nicole Ferraro, Benjamin J. Strober, Francois Aguet, Silva Kasela, Marios Arvanitis, Bohan Ni, Laurens Wiel, Elliot Hershberg, Kristin Ardlie, Dan E. Arking, Rebecca L. Beer, Jennifer Brody, Thomas W. Blackwell, Clary Clish, Stacey Gabriel, Robert Gerszten, Xiuqing Guo, Namrata Gupta, W. Craig Johnson, Tuuli Lappalainen, Henry J. Lin, Yongmei Liu, Deborah A. Nickerson, George Papanicolaou, Jonathan K. Pritchard, Pankaj Qasba, Ali Shojaie, Josh Smith, Nona Sotoodehnia, Kent D. Taylor, Russell P. Tracy, David Van Den Berg, Matthew T. Wheeler, Stephen S. Rich, Jerome I. Rotter, Alexis Battle, Stephen B. Montgomery

**Affiliations:** 1 Department of Biomedical Engineering, Johns Hopkins University School of Medicine, Baltimore, MD, USA; 2 Biomedical Informatics Training Program, Stanford University, Stanford, CA, USA; 3 Harvard School of Public Health, Epidemiology Department, Boston, MA, USA; 4 Broad Institute of MIT and Harvard, Cambridge, MA, USA; 5 New York Genome Center, New York, NY, USA; 6 Department of Systems Biology, Columbia University, New York, NY, USA; 7 Department of Medicine, Division of Cardiology, Johns Hopkins School of Medicine, Baltimore, MD, USA; 8 Department of Computer Science, Johns Hopkins University, Baltimore, MD, USA; 9 Division of Cardiovascular Medicine, Department of Medicine, Stanford University School of Medicine, Stanford, CA, USA; 10 Department of Genetics, Stanford University, Stanford, CA, USA; 11 McKusick-Nathans Institute, Department of Genetic Medicine, Johns Hopkins University School of Medicine, Baltimore, MD, USA; 12 National Heart, Lung, and Blood Institute, National Institutes of Health, Bethesda, MD, USA; 13 Cardiovascular Health Research Unit, Departments of Medicine and Epidemiology, University of Washington, Seattle, WA, USA; 14 Department of Biostatistics, School of Public Health, University of Michigan, Ann Arbor, MI, USA; 15 Cardiovascular Institute, Beth Israel Deaconess Medical Center, Harvard Medical School, Boston, MA, USA; 16 The Institute for Translational Genomics and Population Sciences, Department of Pediatrics, The Lundquist Institute for Biomedical Innovation at Harbor-UCLA Medical Center, Torrance, CA, USA; 17 Collaborative Health Studies Coordinating Center, University of Washington, Seattle, WA, USA; 18 Department of Medicine, Duke University School of Medicine, Durham, NC, USA; 19 Department of Genome Sciences, University of Washington, Seattle, WA, USA; 20 Department of Genetics and Biology, Stanford University, Palo Alto, CA, USA; 21 Department of Biostatistics, University of Washington School of Public Health, Seattle, WA, USA; 22 Laboratory for Clinical Biochemistry Research, University of Vermont, Burlington, VT, USA; 23 Department of Preventive Medicine, University of Southern California, Los Angeles, CA, USA; 24 Center for Public Health Genomics, University of Virginia, Charlottesville, VA, USA; 25 Malone Center for Engineering of Healthcare, Johns Hopkins University, Baltimore, MD, USA; 26 Department of Pathology, Stanford University, Stanford, CA, USA

**Keywords:** rare variants, multi-omics, functional genomics, machine learning, transcriptome, proteome, methylome

## Abstract

Each human genome has tens of thousands of rare genetic variants; however, identifying impactful rare variants remains a major challenge. We demonstrate how use of personal multi-omics can enable identification of impactful rare variants by using the Multi-Ethnic Study of Atherosclerosis, which included several hundred individuals, with whole-genome sequencing, transcriptomes, methylomes, and proteomes collected across two time points, 10 years apart. We evaluated each multi-omics phenotype’s ability to separately and jointly inform functional rare variation. By combining expression and protein data, we observed rare stop variants 62 times and rare frameshift variants 216 times as frequently as controls, compared to 13–27 times as frequently for expression or protein effects alone. We extended a Bayesian hierarchical model, “Watershed,” to prioritize specific rare variants underlying multi-omics signals across the regulatory cascade. With this approach, we identified rare variants that exhibited large effect sizes on multiple complex traits including height, schizophrenia, and Alzheimer’s disease.

## Highlights

• Multi-omics outlier signals inform functional rare variation
• Discovery of large-effect rare variants in a large multi-ancestry cohort
• Watershed prioritizes impactful rare variants for each outlier signal
• Multi-omics outliers can aid identification of causal rare disease genes

## Graphical abstract

**Figure:**
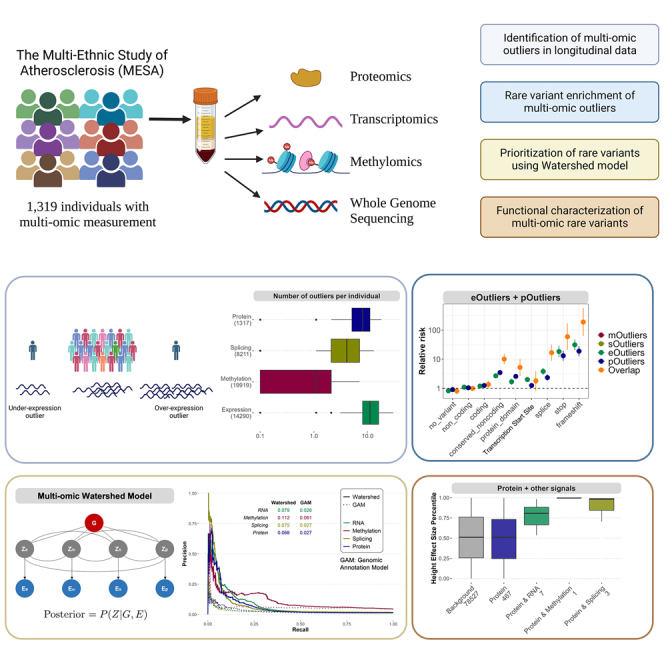

## Teaser

Li et al. investigate the functional impact of rare genetic variation across the regulatory cascade from RNA to protein. Using multi-omics data from diverse individuals, they demonstrate that rare variants are enriched in multi-omics outlier signals and present a flexible framework, Watershed, to prioritize large-effect rare variants underlying each outlier signal. This framework can catalyze the interpretation of rare variants and the discovery of rare disease genes.

## Introduction

There are thousands of rare (minor allele frequency [MAF] < 1%) genetic variants in every human genome but determining which, if any, exert a significant phenotypic effect remains challenging. Prior work has demonstrated the use of transcriptome data in prioritizing rare variants with both large molecular and phenotypic effects.^1^^,^^2^ However, rare variants have the potential to influence additional regulatory mechanisms beyond transcription, such as DNA methylation and protein expression, and integrating corresponding functional genomics data can allow for more comprehensive detection of impactful rare variants and understanding of their roles in the regulation of gene function.

The ability of transcriptome data to enhance prioritization of rare variants with effects on diseases and traits^3^ is presumably due to those effects propagating through the regulatory cascade to protein levels and cellular functions. Prior work has shown that common variants associated with changes in gene expression can have effects on ribosome and protein levels, although those effects are significantly reduced at the protein level.^4^^,^^5^ We and others have also shown that common variants can be associated with changes in protein abundance yet not show any impact at the mRNA level, indicating the effects of post-translational regulation, in addition to the substantial effects of post-transcriptional and protein degradation regulation.^4^^,^^5^^,^^6^ In particular, the plasma proteome contains proteins generated from many different cell types, leading to its regular use as a source for biomarker discovery^6^^,^^7^; therefore, understanding how rare genetic variation impacts protein abundance in samples such as plasma may help identify impactful rare variants from tissues that are more challenging to transcriptome sequence.^8^

In this study, we expand the assessment of impactful rare variation to integrate molecular signatures across the regulatory cascade. We analyzed measurements of DNA methylation from whole blood, RNA sequencing from peripheral blood mononuclear cells (PBMCs), and plasma proteome abundance from a multi-ethnic cohort of ∼900 individuals with data from two time points 10 years apart, and assessed the ability of each measurement to prioritize nearby rare variation. Notably, we observed that the longitudinal design of these data provided robust outlier measurements per individual per data modality. We subsequently integrated these diverse functional signals into a predictive model to assign probabilities to individual rare variants leading to functional effects at various levels of the regulatory cascade. Finally, we demonstrated the utility of these predicted functional probabilities in prioritizing variants with large effects on downstream traits and diseases.

## Results

### Consistency of outlier measurements across time

**Figure 1:**
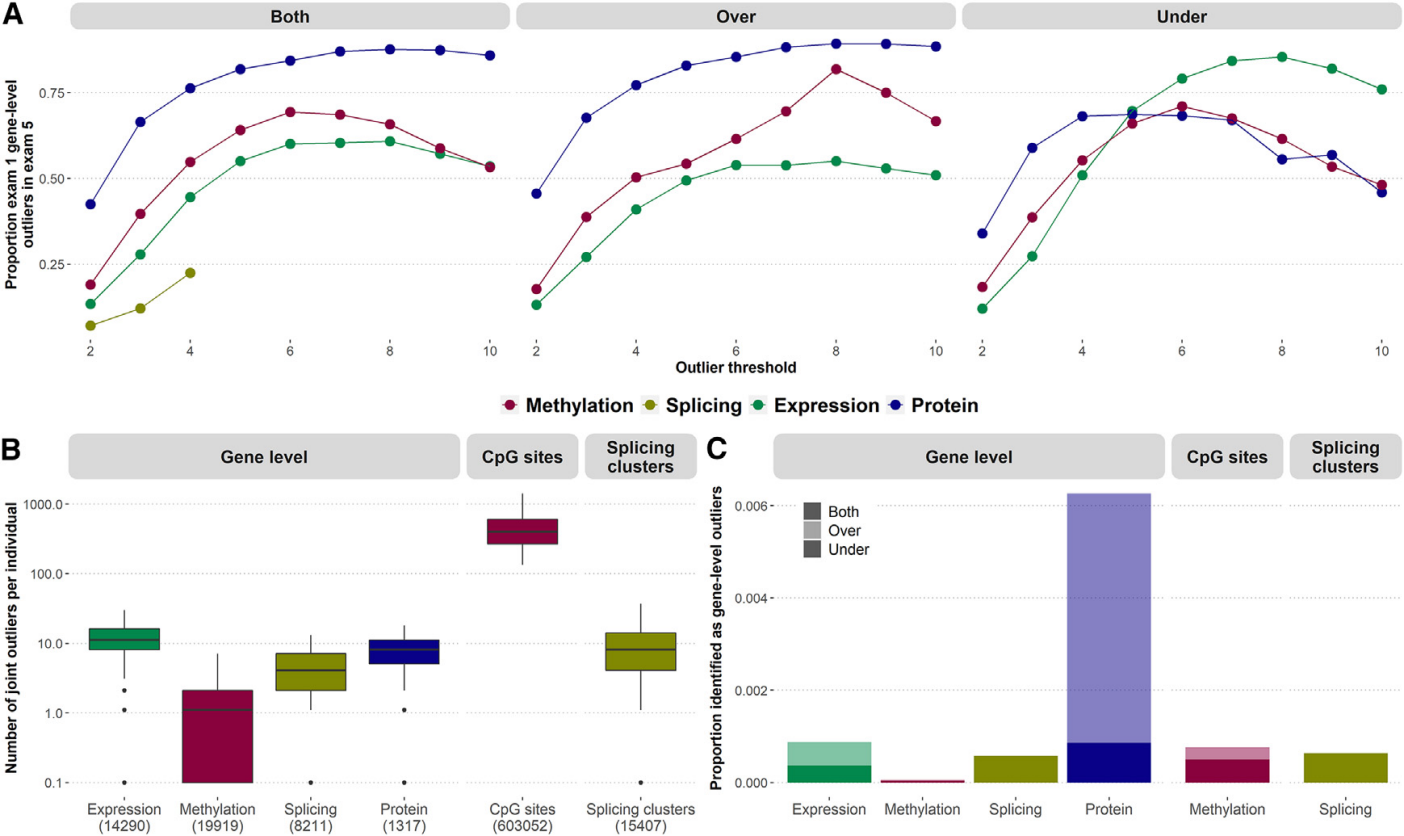
Outlier calls across exams (A) Proportion of gene-level outliers identified in exam 1 (y axis) at varying thresholds (x axis) that replicate in exam 5 at a threshold of |*Z*| > 2. sOutliers (gold) do not have direction and so are shown only for the combined set of outlier calls (left), while eOutliers (green), mOutliers (red), and pOutliers (blue) are also shown split by direction, with outliers with positive *Z* scores in the center (“Over”) and negative *Z* scores on the right (“Under”). (B) Number of outliers identified per individual where the outlier effect is seen in both exams, using a threshold of |*Z*| > 3 for all gene-level outliers (left), as well as the number of CpG-level mOutliers (center) and sOutlier clusters (right). Boxplots represent median and interquartile range. (C) Proportion of all gene-individual pairs considered that show outlier signal in both exams, using a threshold of |*Z*| > 3, split by the direction of the effect.

From a total of 1,319 participants in the Multi-Ethnic Study of Atherosclerosis (MESA) cohort with whole-genome sequencing, we uniformly processed transcriptomic, methylomic, and proteomic data for each gene in each individual after controlling for known and hidden covariates, including genotype PCs, to calculate residual *Z* scores. We defined outliers as those (gene, individual) pairs which reach *Z-*score threshold of either 2 or 3 depending on the context of our analysis. These outliers represent levels of measured molecular signals that are significantly higher (over-outliers) or lower (under-outliers) compared to population mean. Assessing the correlation of multi-omics measurements across participants between 10-year time points of collection, plasma proteome measurements exhibited the highest correlation (median Pearson correlation coefficient r = 0.67), followed by expression (median r = 0.24), gene-level methylation (median r = 0.16), and gene-level splicing (median r = 0.05) (Figure S1). Almost all measured proteins showed significant correlation between the two exams at Bonferroni-adjusted p value threshold of 0.05 (99.7%), followed by gene expression (60.6%), gene-level methylation (46.5%), and gene-level splicing (10.5%). We then assessed replication across time for the subset of measurements at the extremes of the distribution (“outliers”) for each gene-level outlier type. We refer to those instances for gene expression as “eOutliers,” methylation as “mOutliers,” splicing as “sOutliers,” and protein as “pOutliers.” After identifying outliers in exam 1, based on an individual’s deviation from the mean for a given gene (*Z* score), we assessed the proportion that also had measurements at least two standard deviations from the mean in exam 5. Across thresholds, we observed the highest replication for pOutliers (range 0.34–0.89), followed by mOutliers (range 0.18–0.82), eOutliers (range 0.12–0.85) and sOutliers (range 0.07–0.22). When focusing on the subset of eOutliers with negative *Z* scores and thus very low expression, we saw the replication rate across time increasing with threshold stringency (Figure 1A), eventually surpassing all other replication rates when the measurements were over ∼6 standard deviations below the mean (*Z* < −6), at which point 79% of exam 1 eOutliers were also seen in exam 5. This is in line with prior work demonstrating that underexpression outliers are more often associated with rare variants and are thus likely more often genetically driven than overexpression outliers.^1^^,^^2^ To focus on robust and more likely genetically driven outlier events, we took advantage of the longitudinal study design and required an outlier effect to be seen in both time points in subsequent analyses (“joint outliers”). For joint outliers, we observed an average of 12.5 eOutliers, 1.2 gene-level mOutliers (472 CpG-level mOutliers), 4.8 sOutliers (9.9 sOutlier clusters), and 8.3 pOutliers per individual (Figure 1B).

We assessed the relative proportion of joint outlier events for each omics data type. Restricting the analysis to individuals with data in both time points, we assessed 14,290 genes across 547 individuals for eOutliers, 19,919 genes across 785 individuals for mOutliers, 8,211 genes across 564 individuals for sOutliers, and 1,317 proteins across 876 individuals for pOutliers. Looking at the proportion of tests that resulted in joint outliers at a threshold of |*Z*| > 3, we found the highest proportion for pOutliers, followed by eOutliers (Figure 1C), consistent with the observation that pOutliers demonstrated highest correlation across exams. Overall, the set of pOutliers contained many more high-abundance outliers compared to low-abundance outliers, while the proportions in either direction were more comparable for eOutliers and mOutliers. This could reflect the dynamic range of the protein measurements, as previous work has found the range of protein abundances detectable to be higher than that of mRNA transcripts,^9^ and there is no strict upper bound for high-abundance outliers, while detected protein abundances can only decrease to 0. We further found that the number of high- and low-abundance pOutliers discovered varies by protein type (Figure S2A) or inferred tissue of origin (Figure S2B) and observed that classes of proteins with higher base expression tended to have more low-abundance pOutlier individuals than the set of all proteins and vice versa (Figures S2C and S2D).

### Outlier sharing across the regulatory cascade

**Figure 2:**
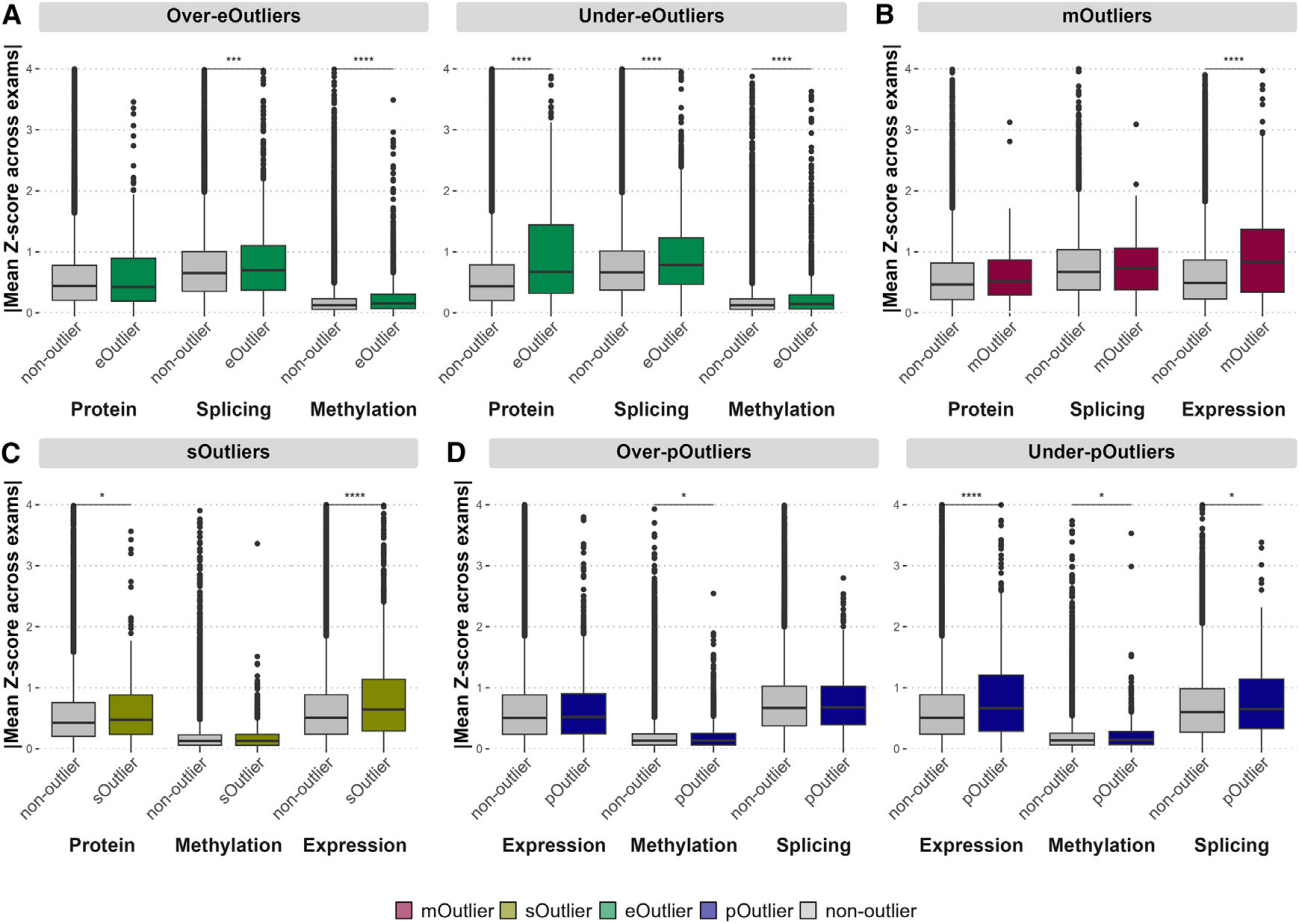
Distribution of *Z* scores for outliers in other data types (A) Distribution of gene-level methylation, gene-level splicing, and protein *Z* scores for eOutlier individuals (green) and non-outliers (gray) for corresponding genes, split by the direction of the expression effect. (B) Distribution of expression, gene-level splicing, and protein *Z* scores for mOutlier individuals (red) and non-outliers (gray) for corresponding genes. (C) Distribution of expression, gene-level methylation, and protein *Z* scores for sOutlier individuals (gold) and non-outliers (gray) for corresponding genes. (D) Distribution of expression, gene-level methylation, and gene-level splicing *Z* scores for pOutlier individuals (blue) and non-outliers (gray) for corresponding genes, split by the direction of the expression effect. ∗∗∗∗p < 0.0001, ∗p < 0.05, one-sided Wilcoxon rank-sum test on absolute value of mean *Z* score across both exams between outlier and non-outlier individuals.

While each data type was measured in different biospecimens with DNA methylation from whole blood, expression data from PBMCs, and protein measurements from plasma, we assessed the sharing of outlier signals across each omics data type, as rare-variant effects can manifest across multiple tissues.^1^^,^^2^ For the set of joint outliers identified in each data type at a threshold of |*Z*| > 3, we assessed the mean *Z* scores across exams in all other gene-level data types. For under-eOutlier individuals, there were significant shifts in corresponding methylation (p = 1.5e−15, one-sided Wilcoxon rank-sum test), splicing (p < 2.2e−16), and protein (p = 5.1e−14) *Z* scores. For over-eOutlier individuals, there were significant shifts in methylation (p < 2.2e−16) and splicing (p = 2.5e−5) *Z* scores for over-eOutliers (Figure 2A). For gene-level mOutlier and sOutlier individuals, there was a significant increase in the corresponding expression *Z* scores (p = 2.8e−13 and p < 2.2e−16, respectively; Figures 2B and 2C). For low-abundance pOutlier individuals, there was a corresponding significant shift in expression values (p = 1.1e−11), although this is not the case for high-abundance pOutlier individuals (Figure 2D).

For eOutliers, the highest degree of sharing was seen at the protein level. Of 485 eOutliers (|*Z*| > 2 in both exams) identified in genes and individuals that also had protein measurements, we found that 12% of those (N = 58) were shared at the protein level, with 29.3% (N = 17) of those being high-abundance pOutliers. For all other gene-level outlier types (mOutliers, sOutliers, and pOutliers), the highest degree of sharing was seen at the expression level, with 18.2%, 8.9%, and 3.7% of mOutliers, sOutliers, and pOutliers, respectively (Figure S3A). Considering only under-eOutliers, 20.8% of those showed outlier protein levels and for low-abundance pOutliers; 15.1% also had outlier expression levels (Figure S3B). Notably, outlier signals replicate best with consistent directions (e.g., underexpression with underprotein levels, Figure S3C). Overall, eOutliers had the strongest shift in values for other functional measurements and best captured outlier signals across all other data types, particularly when the outlier effect led to very low expression, indicating that the transcriptome best captured effects that propagated throughout the regulatory cascade, although any one individual measurement does not capture all instances of potentially abnormal function.

### Rare variants contribute to outlier effects across multi-omics data types

We expect rare variants to contribute substantially to the observed outlier effects, as has been thoroughly demonstrated for transcriptome outliers^2^ and investigated for methylation^10^^,^^11^ and protein levels.^12^ As we have multiple omics measurements for the same individuals and observed that a proportion of outlier effects are shared between molecular phenotypes, we sought to assess the degree to which rare variation contributed to each gene-level outlier signal and the benefit of collecting multiple omics measurements for rare-variant interpretation. Here, rare variants were annotated from the MESA cohort as single-nucleotide variants and small insertions and deletions that appear at a less than 1% frequency across MESA as well as across the entire gnomAD dataset and in all relevant gnomAD subpopulations (STAR Methods).

Considering each of the four gene-level outlier types individually, we observed the strongest enrichment for mOutliers, which carried rare variants in the outlier gene body or within 10 kb between 1.11 and 1.55 times as frequently as non-outliers, depending on threshold stringency (|*Z*| threshold between 2 and 4). This was followed by eOutliers (relative risk = 1.10–1.29), sOutliers (relative risk = 1.02–1.26), and pOutliers (relative risk = 1.03–1.06), considering rare variants within the same 10-kb window. pOutliers had the smallest enrichment despite having highest replication across exams; interestingly, a recent study on common variants impacting plasma proteome quantitative trait loci (pQTLs) reported ∼40% of proteins had only *trans*-pQTLs (>500 kb from target), suggesting that protein-impactful rare variants may be more often located in *trans*.^13^ Joint CpG-level mOutliers were strongly enriched for carrying nearby rare variants across windows that ranged from 100 bp (relative risk = 52.9, p < 2.2e−16, one-sided t test) to 1 kb (relative risk = 5.84, p < 2.2e−16) around the site. These enrichments were largely driven by instances where rare variants overlapped the CpG site itself but remain significant after removal (Figures S4A and S4B). As a further signature of a rare-variant effect, CpG-level mOutliers also showed a significant increase in allele-specific expression in a 1-kb window around outlier sites (Figure S4C). When considering mOutliers in different genomic regions relative to CpG islands and promoters, we found that each individual has a slightly higher fraction of outliers located within CpG islands compared to adjacent regions, and a lower fraction of promoter-associated mOutliers compared to intergenic regions (Figure S5A). While we did not observe differences in rare-variant enrichment for mOutliers located near the transcription start site (TSS) or not, we found that hypomethylation outliers had a higher enrichment for CpG island probes, whereas hypermethylation outliers had a higher enrichment for CpG shelf (Figures S5B and S5C).

**Figure 3:**
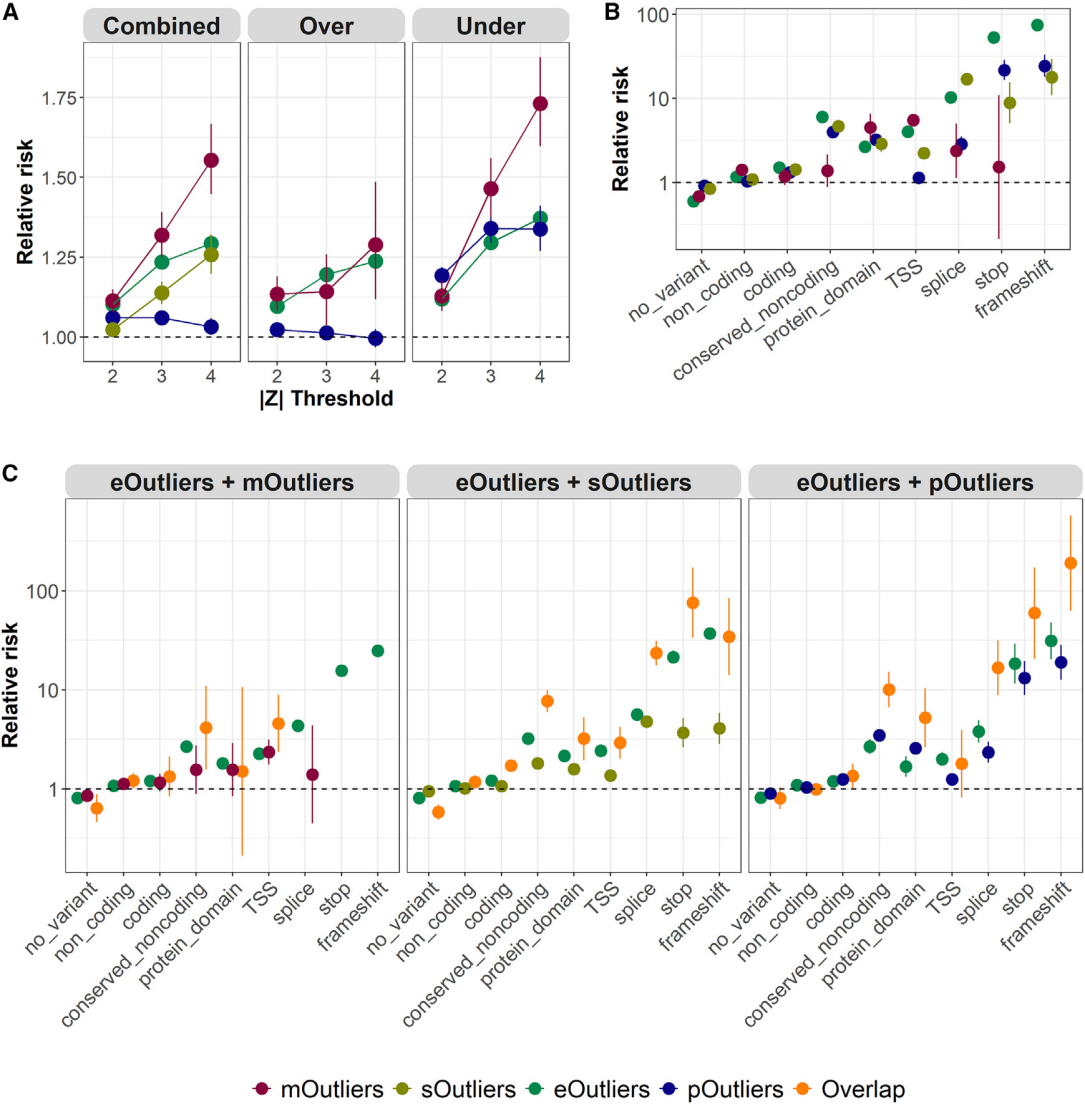
Enrichment of rare variants near gene-level outliers (A) Relative risk of nearby rare variants for eOutliers (green), gene-level mOutliers (red), gene-level sOutliers (gold), and pOutliers (blue) across varying *Z* score thresholds (x axis). Enrichments are split by the direction of the effect for eOutliers, mOutliers, and pOutliers. Non-outliers are defined as all individuals with |*Z*| < 1 in both exams for the same set of genes. (B) Relative risk of nearby rare variants with a given annotation (x axis) for eOutliers (green), gene-level mOutliers (red), gene-level sOutliers (gold), and pOutliers (blue) at a threshold of |*Z*| > 3 in both exams. Non-outliers are defined as all individuals with |*Z*| < 1 in both exams for the same set of genes. If no outlier individual carried a nearby rare variant in a given category, that data type is not shown. (C) Relative risk of nearby rare variants with a given annotation (x axis) for combinations of eOutliers and the other three data types (orange) as compared to single data type outliers, matched for the same considered genes and individuals, considering a reduced threshold of |*Z*| > 2 in both exams, and in both data types for the overlapping set. Non-outliers are defined as all individuals with |*Z*| < 1 in both exams for the same set of genes. If no outlier individual carried a nearby rare variant in a given category, that data type is not shown. Error bars represent 95% confidence interval of enrichment.

Previous studies have observed stronger rare-variant enrichments for under-eOutliers compared to over-eOutliers.^2^ For expression, we observed similar patterns (relative risk = 1.12–1.37 for under-eOutliers and 1.10–1.24 for over-eOutliers, where comparing enrichment between under-eOutliers and over-eOutliers resulted in *p* < 5e−4 for outlier threshold between 2 and 4, two-sided t tests). This observation also held for other omics data types included in our study. When splitting by the direction of the effect for methylation, hypomethylated mOutliers (relative risk = 1.13–1.73) showed stronger enrichments than hypermethylated mOutliers (relative risk = 1.13–1.29, p < 1.9e−4 when comparing enrichment between hypomethylated mOutliers and hypermethylation mOutliers for outlier threshold between 3 and 4). Likewise, for pOutliers we observed higher enrichment for under-outliers (relative risk = 1.19–1.34) compared to over-outliers (relative risk = 0.99–1.02, p < 1e−22 for outlier threshold between 2 and 4). Notably, high-abundance pOutliers were not significantly enriched for nearby rare variation at any threshold above |*Z*| > 2 (relative risk = 1.02, p = 1.54e−3 at |*Z*| = 2; non-significant at |*Z*| > 2) (Figure 3A). This lack of enrichment was not entirely due to the restricted set of genes assayed for protein abundance, as restricting the set of eOutliers to the genes also assayed at the protein level still resulted in significant enrichments for nearby rare variants in the overexpression direction at thresholds of |*Z*| > 2 (relative risk = 1.10, p = 1.97e−8) and |*Z*| > 3 (relative risk = 1.11, p = 0.013) (Figure S6).

We next evaluated whether different categories of rare variants contributed to observed enrichments in each omics data type. Considering different predicted effects across variants, eOutliers were most strongly enriched for nearby rare stop and frameshift variants, as expected based on previous work,^2^ while sOutliers were most strongly enriched for nearby rare splice variants, with strong enrichments also for rare stop and frameshift variants. For gene-level mOutliers most variant categories were not significantly enriched, but rare variants near the associated gene’s TSS were seen 5.49 times (p < 2.2e−16, one-sided t test) as frequently in mOutlier individuals compared to controls (Figure 3B). While the combined sets of pOutliers were largely not enriched for nearby rare variants overall, there was strong enrichment for nearby rare stop (relative risk = 21.7, p < 2.2e−16) and frameshift (relative risk = 24.4, p < 2.2e−16) variants, although this was predominantly driven by underexpression pOutliers (Figure S7).

### Multi-omics outliers increase discovery of rare-variant effects

As expression outliers best captured outlier signals in other data types (Figures 2 and S3), we assessed the gain in rare-variant enrichments when considering eOutliers in conjunction with outliers for each of the other data types. While for many types of variants eOutliers alone tagged functional rare variants at a frequency similar to that of the set of multi-modal outliers, there was a subset of variant types for which multi-omics data improved functional rare-variant identification (Figure 3C). Most notably, the set of gene-individual pairs that showed outlier signal at both the expression and protein level are more strongly enriched for nearby rare conserved non-coding (p < 4.1e−7, one-sided t test), protein-domain region (p < 0.02), splice (p < 1.2e−5), stop (p < 0.02), and frameshift variants (p < 1.4e−3) as compared to the set of eOutliers or pOutliers identified alone in the same set of genes and individuals. When considering both expression and methylation signals, there was an improvement in enrichment for nearby rare TSS (p < 0.04) variants over either data type alone, and for overlapping expression and splicing signal, the enrichment of nearby rare conserved non-coding variants (p < 2.0e−11) and rare splice (p < 2.7e−21) and stop variants (p < 0.001) were all increased (Figure 3C), indicating that for specific variant effects, assessing multiple molecular signals can improve identification of functional rare variants.

In practice, it may be difficult to collect both multiple omics measurements from an individual as well as data across multiple time points. While we are limited by the relatively smaller number of proteins assayed as compared to gene expression measurements, we assessed the relative gain in enrichments considering both expression and protein outliers identified from only a single time point as compared to outlier effects seen in each specific omics data type measured at two time points. While the set of overlapping eOutliers and pOutliers at a single time point is small (N = 72 at a threshold of |*Z*| > 3), we do see increased enrichment of nearby rare variation (relative risk = 1.37, p = 1.71e−4, one-sided t test) over either joint eOutliers (relative risk = 1.23, p < 2.2e−16) or joint pOutliers (relative risk = 1.06, p = 1.72e−9) at that same threshold or higher (Figure S8). This indicates that multi-omics measurements are providing enhanced ability to detect rare-variant-driven outliers compared to repeated measures of a specific omics data type over time.

### Replication of GTEx outlier-associated rare variants

Our previous work identified rare variants associated with multi-tissue transcriptome outliers in the Genotype Tissue Expression project (GTEx),^2^ which consisted primarily of individuals of European ancestry. Here, we observed significant correlation between individual outlier burden and genotype principal components (PCs), which decreased at increasing outlier thresholds (Figure S9). Notably, we saw little difference in rare-variant enrichment estimates after matching each outlier individual to a control individual by ancestry, as measured by genotype PCs (Figure S10), and thus did not observe evidence of differences in genetic ancestry driving the observed enrichment of rare variants nearby any outlier type. Next, we evaluated the proportion of those GTEx variants that are carried by any individual in MESA and exhibit consistent effects on gene expression and splicing. For eOutliers, we identified 1,348 multi-tissue eOutlier-associated variants in GTEx that were present in any MESA individual and occurred at <1% frequency across MESA, which totaled 5,604 total variant-gene-individual instances (Figure S11A). Of these, 888 also showed outlier expression in MESA, at a reduced threshold of |*Z*| > 2 in both exams (empirical q < 0.01; Figure S11B and STAR Methods). We found that rare stop variants are most predictive of replicating expression effects, followed by rare splice variants (Figure S11C). For sOutliers, we identified 1,113 multi-tissue sOutlier-associated variants in GTEx that were present in any MESA individual, which totaled 5,858 total variant-gene-individual instances (Figure S12A). Of these, 891 also showed outlier splicing in MESA, at a reduced threshold of |*Z*| > 2 in both exams (empirical q < 0.01; Figure S12B). We observed that rare splice variants are most predictive of replicating outlier splicing effects (Figure S12C).

### Development and evaluation of multi-omics rare-variant prediction model

**Figure 4:**
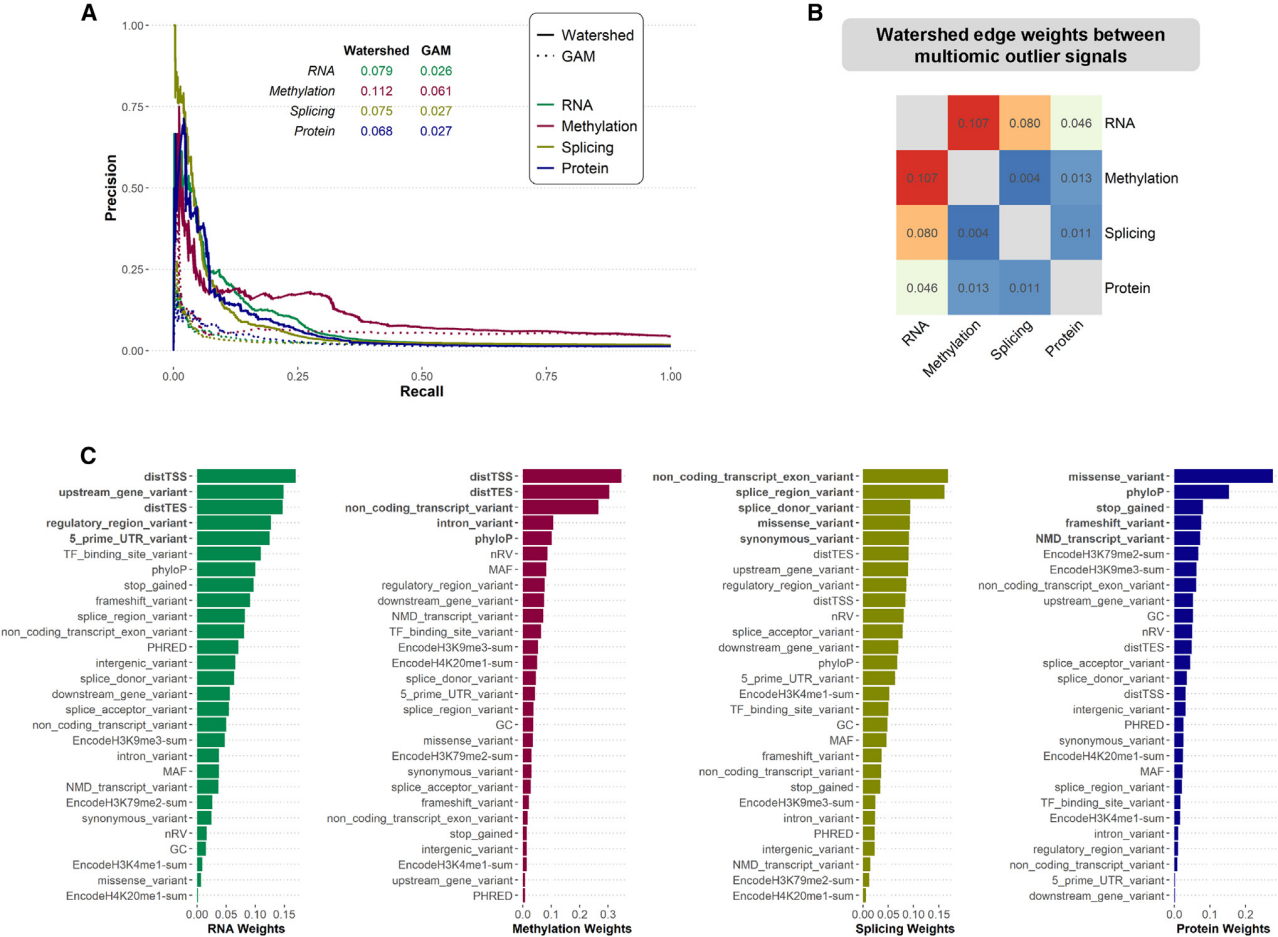
Evaluation of multi-omics Watershed model (A) Precision-recall curves of Watershed models (solid lines) and genomic annotation models (GAM, dotted lines) for mRNA expression (green), methylation (red), splicing (gold), and protein expression (blue) evaluated against (gene, individual) pairs with the same set of rare variants nearby. GAM outliers are defined by a p value threshold of 0.05. (B) Symmetric matrix summarizing weights of edges connecting the latent regulatory variables (*Z*) in the multi-omics Watershed model. (C) Edge weights connecting top genomic annotation features to latent regulatory variables in each omic signal, ranked by the relative informativeness in decreasing order. The top five most influential genomic annotations are shown in bold for each outlier signal. A detailed explanation of each genomic annotation features included in the model is provided in Table S1.

To leverage the full spectrum of data available in MESA to prioritize functional rare variants, we extended our Bayesian hierarchical variant effect prediction model, Watershed.^2^ In brief, Watershed integrates genomic annotations such as conservation scores and variant annotations with observed outlier signals from functional data in a latent variable model originally developed for transcriptomic outliers. Here, we extended Watershed to include mRNA expression, methylation, splicing, and protein expression (Figure S13A). When evaluated against pairs of individuals with the same rare variant near the same gene (N2 pair), the multi-omics Watershed model outperformed logistic regression models based on genomic annotations alone (GAM) in predicting the regulatory status of one individual in the pair based on the genomic annotations and observed outlier status in the other, achieving an area under the precision-recall curve between 0.07 and 0.11 across the four omics data types (Figure 4A), compared to 0.02–0.06 for GAM.

Examining the learned parameters of the multi-omics Watershed model, we observed higher edge weights connecting RNA and methylation, and RNA and splicing signals, compared to those connecting protein and other signals, suggesting varying levels of information sharing between signals in modeling rare-variant effects (Figure 4B). Consequently, the multi-omics Watershed model outperformed corresponding RIVER models, which were trained on single-omic data types at a time, due to information sharing (Figure S13B). The learned weights contributed by each genomic feature also reflect known regulatory biology, with distance-based features being highly informative for RNA and methylation outlier signals, splicing annotations most predictive of splicing outlier signal, and missense and loss-of-function annotations most predictive of protein outlier signals (Figure 4C). These results indicate that our multi-omics Watershed model captures biological signals underlying rare variants’ effect on outlier expression across aspects of the regulatory cascade to jointly prioritize functional rare variants.

Given that we observed little difference in the enrichment of rare-variant burden when considering all individuals or matching by ancestry within MESA (see STAR Methods), we sought to assess the portability of the multi-omics Watershed model across ancestries. We estimated genetic ancestry based on the Human Genome Diversity Panel with seven superpopulations^14^ and assigned population groups by thresholding the proportion of ancestry estimates (Figure S14). We then trained the multi-omics Watershed model using data from N = 426 individuals assigned to European ancestry and evaluated its performance on N2 pair individuals from other populations. We observed comparable predictive performance in terms of area under the precision-recall curve assessment across these populations (Figure S15), suggesting that outlier rare-variant effects discovered in one population are likely to exhibit comparable effects across populations, as expected if we are identifying truly causal variants in the absence of significant non-genetic contributions.

### Multi-omics prioritized rare variants are prevalent in each individual

**Figure 5:**
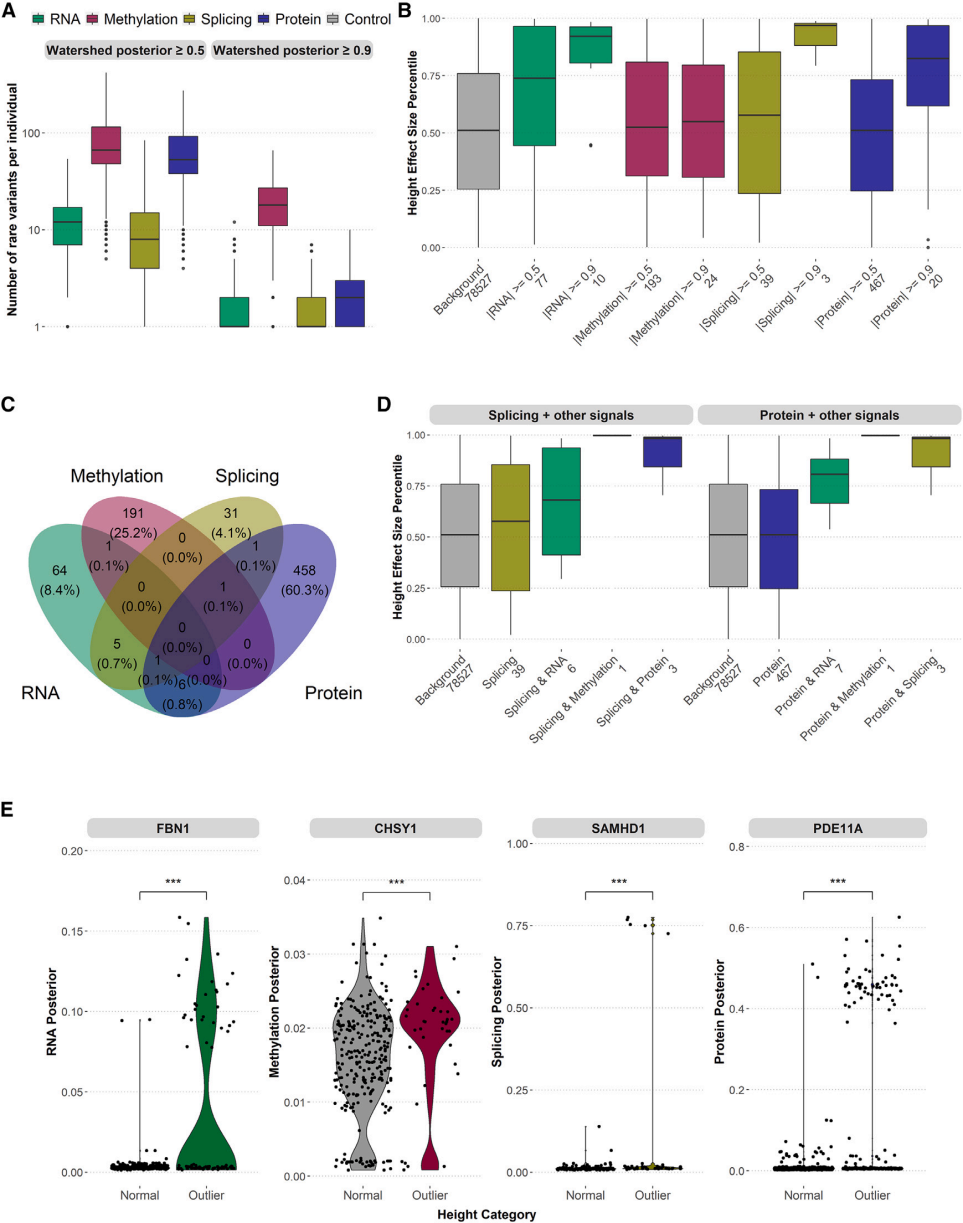
Application of multi-omics Watershed model to inform trait associations (A) Number of rare variants per individual as prioritized by each omic signal (mRNA expression, green; methylation, red; splicing, gold; protein expression, blue) at two levels of Watershed posterior cutoff 0.5 and 0.9. Individuals with significantly large number of outlier expressions (“global outliers”) are removed. The y axis is transformed to log scale. Boxplots represent median and interquartile range. (B) Distribution of percentile normalized effect size for height (median and interquartile range) of all rare variants (background, gray), and those rare variants prioritized by multi-omics Watershed in each signal at two posterior threshold values. Only rare variants mapped to genes with evidence of causing abnormal body height as reported by the Human Phenotype Ontology (HPO) are shown (N = 1,314 genes). Number of rare variants in each category is shown in x axis labels. Effect-size estimate was obtained from UK Biobank GWAS Round 2 using rank-normalized phenotype. (C) Venn diagram of rare variants prioritized by each signal at a posterior threshold of 0.5. (D) Distribution of percentile normalized effect size for height (median and interquartile range) for rare variants prioritized by a single signal at a posterior threshold of 0.5 (left, splicing; right, protein expression) and combined with another signal. (E) Distribution of Watershed posteriors of rare variants identified in individuals with normal height (|*Z*| < 0.2) and abnormal height (|*Z*| > 2) in the MESA cohort, collapsed to each gene. Shown are examples of top genes showing differential distribution of posteriors in each omic signal which overlap with HPO annotated genes. ∗∗∗p < 0.001, one-sided Wilcoxon rank-sum test on absolute value of posteriors between normal and outlier individuals.

To assess the individual relevance of the rare variants prioritized by the multi-omics Watershed model, we first observed that each individual’s genome had a significant number of rare variants with large posteriors in each omics data type, with 11 RNA variants, 7 splicing variants, 17 methylation variants, and 52 protein variants with posterior ≥0.5 (Figure 5A). Methylation and protein had the highest number of rare variants with predicted large effects at posterior threshold of 0.5 and 0.9. Strikingly, variants prioritized by different outlier signals were largely non-overlapping (Figure S16A), indicating that multi-omics measurements provided complementary information in characterizing effects of rare variants inaccessible to one omics data type alone, as also supported by the increasing enrichment of nearby rare variants when outlier signals are seen at multiple levels.

To further characterize variants prioritized by each omics data type within an individual, we assessed the probability of loss-of-function intolerance (pLI) scores^15^ for genes mapped to variants in each group. Notably, pLI scores were not included as an annotation in the Watershed model. We observed that genes with large-effect variants across multiple signals tended to have lower pLI scores and thus were more tolerant of damaging mutations (Figure S16B). Moreover, when we systematically annotated Watershed-prioritized variants with Pfam protein domain information^16^ from MetaDome,^17^ we found that variants located at evolutionary equivalent (i.e., meta-domain) positions had higher posteriors in all outlier signals (Figure S16C, p < 2.2e−16, one-sided Wilcoxon rank-sum test). Overall, these data suggest that multi-omics Watershed posteriors capture functional impact of rare variants and provide a strong basis for the application of Watershed posteriors to inform trait associations.

### Multi-omics prioritized rare variants impact multiple complex traits and diseases

We sought to test the hypothesis that rare variants with large multi-omics-based Watershed posteriors are likely to be causal for traits and diseases. We first focused on height, a highly polygenic and heritable trait collected for all individuals in MESA. Based on the summary statistics estimated from a separate cohort, UK Biobank, we identified 78,527 rare variants that overlap with the scored variants in our multi-omics Watershed model which maps to 1,314 genes known to cause abnormal body height as cataloged in the Human Phenotype Ontology (HPO^18^). When restricting to variants prioritized by Watershed with posterior >0.5 or >0.9, we observed higher effect sizes on height as compared to background (Figures 5B and S17). The observed higher effect sizes were robust to selecting only the top N variants (N = 10 and 100) from each data type separately (Figure S18A).

Notably, multi-omics outliers could prioritize Mendelian or large-effect genes. We identified a small set of rare variants which were mapped to Mendelian height genes with posterior >0.5 or were among the top 100 highest posteriors in more than one signal (Figures 5C and S18B). However, when comparing rare variants prioritized by two signals, we found that they had even higher effect sizes compared to those prioritized by single signals, which is especially prominent with splicing and protein when combined with another signal (Figures 5D, S18C, and S18D). Importantly, the shift in effect size by Watershed-prioritized variants was also higher than single-omic outliers further stratified by MAF (Figure S19), suggesting that the functional correlation between Watershed posterior and effects on trait is not solely driven by MAF. These data support the utility of collecting multi-omics measurements from the same individuals to improve prioritization of functional rare variants with large trait effects that could potentially be missed by traditional approaches such as genome-wide association studies (GWASs).

We next assessed whether Watershed-prioritized rare variants could be applied to enhance gene prioritization for complex traits. We obtained body height data on N = 4,559 MESA individuals and, after correcting for known covariates, identified those with average body height (“control” individuals, residual |*Z*| < 0.2) and those with the extremes of body height (“outlier” individuals, residual |*Z*| > 2). When comparing the distribution of posteriors for all rare variants mapped to each gene between outlier and control individuals, we were able to recapitulate known Mendelian height genes (Figure 5E); importantly, different signals prioritized different genes, which further highlighted the complementary nature of each omics data type. When we combined posteriors across all outlier signals for each variant and compared the resulting gene-level p values with other gene prioritization methods based on common variants (MAGMA^19^), rare coding variants (burden test^20^), or expression QTLs (PrediXcan^21^), we found that our approach is largely independent (Figure S20), suggesting the unique advantage of incorporating non-coding rare variants in a gene prioritization framework.

To demonstrate the utility of multi-omics prioritized rare variants with Watershed to a diverse range of traits and diseases, we applied similar analyses to immunological and neuropsychiatric traits. Here, we referenced a recent machine-learning method that systematically characterized causal genes to primarily focus on rare variants impacting genes with well-predicted trait relevance.^22^ For rheumatoid arthritis (RA), we identified 41,339 rare variants with effect-size estimates in GWASs and observed that RNA posteriors strongly correlated with effect size (median rank-normalized effect size = 0.58 for RNA posterior prioritized rare variants compared to 0.50 for background). We further observed that for RA, the protein signal by itself did not prioritize rare variants with large effect size; however, it did when combined with RNA or methylation signals (Figures S21A and S21B). This observation held true for COVID-19 severity, another immunological trait (Figures S21C and S21D). For Alzheimer’s disease (AD), methylation outliers were most predictive of effect size, but multi-modal outliers have much higher impacts (Figures S22A and S22C). Interestingly, joint underexpression outliers in RNA and protein signals identified genes with established associations with AD, such as *PDGFRB*^23^ (rs116171826, rs149274963, and rs10071918), *PTN*^24^ (rs61735090), and *MPO*^25^ (rs35897051), supporting the potential role for our prioritized variants in AD pathobiology. For schizophrenia (SCZ), even though we had a smaller set of rare variants with effect-size estimates (N = 2,851), we observed moderate effect size for variants prioritized by RNA, methylation, and protein signals and a strong shift in splicing prioritized variants (Figure S22D). Notably, in addition to referencing external databases for identifying relevant causal genes, we applied MAGMA to prioritize genes using GWAS summary statistics. We identified 5,378 genes with MAGMA *Z* > 2 (schizophrenia “positive” genes) and 4,092 genes with MAGMA *Z* < 0 (schizophrenia “control” genes), and when we compared rare variants with large Watershed posteriors mapped to these two groups of genes, we observed a significant shift in effect size only within positive genes (Figure S22E). Overall, these analyses demonstrate that the multi-omics Watershed model represents a flexible framework, which can be easily integrated into pipelines for connecting variants to traits.

## Discussion

Rare genetic variants are collectively abundant in the human genome due to recent population expansion.^26^^,^^27^ They are often population private, unlike common variants, which are shared across populations.^28^ Although rare variants have in general larger functional effects on molecular phenotypes that can contribute to the risk of complex diseases,^29^^,^^30^ their abundance may lead to false-positive associations and thus require careful methods for analysis and interpretation.^31^^,^^32^ The present study extends efforts to identify large-effect rare variants through analysis of functional genomics data.^1^^,^^2^^,^^33^^,^^34^^,^^35^^,^^36^^,^^37^ By integrating longitudinal multi-omics data collected from a diverse cohort with matched whole-genome sequencing, we identified significant enrichment of rare-variant burden nearby multi-omics outlier signals across the regulatory cascade.

Our study benefited from both multi-omics data generation and a study design including functional measurements at two time points approximately 10 years apart. We observed higher enrichment of rare-variant burden in multi-modal outliers collected at a single time point compared to joint outliers across two visits based on only a single molecular signal, which indicates that multi-omics datasets can be more beneficial than collecting the same measurement over multiple time points when using those measurements to prioritize functional rare variants. Of interest, while evidence of multi-omics signatures improved rare-variant discovery beyond repeat measurements of any single-omic data type alone, our work also highlighted areas of discordance between multi-omics outliers that may be due to buffering or unknown technical effects.

Importantly, we conducted analyses across an ancestrally diverse cohort, including individuals with genetic ancestry from several major (sub-)continents, namely of African, East Asian, European, and American descent, which allows for the evaluation of many additional rare variants than would be included in a cohort containing individuals with all predominantly European ancestry, as is often the case in genomics research due to the over-representation of European populations.^38^^,^^39^^,^^40^ We found that rare-variant enrichments nearby outliers did not change when comparing against all other control individuals as opposed to restricting to controls with similar ancestry, as has been done in previous studies.^1^^,^^2^ We also found that rare variants associated with multi-tissue expression or splicing changes in GTEx, which consists predominantly of individuals of European ancestry, 15.8% and 15.2% replicated in MESA, which was many more than seen after permuting expression and splicing values. The variants discovered in GTEx that are associated with similar transcriptomic effects in MESA were enriched for rare stop and splice annotations, supporting the use of both genomic annotations and functional signals in variant prioritization.

We extended a Bayesian hierarchical variant effect prediction model, Watershed, to synthesize genomic annotations with observed outlier status in four omics data types. Using multi-omics Watershed, we predicted the functional impact of more than 30 million rare variants and observed that each person in MESA harbors a significant number of rare variants with large posterior probabilities of functional effect. Using this approach, we prioritized multiple novel and known rare variants across common and complex traits and disease including height, RA, COVID severity, AD, and SCZ. Further, we demonstrated how integration of this expanded set of prioritized rare variants aids detection of causal genes.

The performance of the multi-omics Watershed model showed good cross-population portability when trained on European individuals and evaluated in other populations. Because a significant fraction of predicted causal loci underlying complex traits are shared across populations,^41^ our results support the integration of functional measurements to pinpoint variants and biological pathways to improve genetic risk prediction for individuals of diverse ancestry backgrounds and complement recent efforts to improve cross-population portability of polygenic risk scores through predicted gene expressions using common variants.^42^ Our data provide compelling evidence that the Watershed model offers a flexible framework for integrative analysis of multi-omics data from diverse populations. Future research can more rigorously evaluate the consistency of rare-variant regulatory mechanisms across populations.

Our work complements the growing literature in connecting common genetic variation to traits through molecular QTLs.^43^^,^^44^^,^^45^^,^^46^^,^^47^^,^^48^^,^^49^ It supports a unified framework where multi-omics functional signals can inform prioritization of genetic variation across the entire frequency spectrum, such that the incorporation of rare variants into common variant frameworks such as polygenic risk scoring could improve stratification of patient risks.^3^ An important consideration when predicting disease risk using Watershed posteriors on rare variants, however, is the relevant genetic regulatory context. Our current model was trained using multi-omics data collected from blood samples from healthy donors, which may serve as a reasonable background for immunological traits and the two neuropsychiatric diseases we considered (AD and SCZ). It remains unclear to what extent rare-variant effects manifest across different tissue contexts. A multi-omics Watershed model trained on data from other tissues and evaluated across a broader range of traits may shed light on trait-specific optimal models for prioritization of rare variants. On the other hand, given the heterogeneous nature of omics data collected from bulk tissues, Watershed models trained on single-cell multi-omics outlier signals may improve our understanding of context-specific regulatory mechanisms and further improve the power to detect functional rare variants.^50^

Our current study showed proof-of-concept analysis applying Watershed posteriors for gene-level association tests, which recapitulated known Mendelian height genes. Although conceptually simple, this framework has several key advantages. First, we observed that multi-modal posteriors provided complementary information and prioritized a largely non-overlapping set of rare variants. When performing gene associations, this method can inform actionable hypotheses about molecular mechanisms underlying top candidates. Second, because we were able to analyze all rare variants in individuals with multi-omics measurements, we assigned posterior probabilities to a large number of non-coding rare variants. Incorporating these non-coding variants may significantly boost power for rare-variant association studies.^51^ On a practical note, in large biobank-scale data, once Watershed is trained on a subset of individuals with multi-omics measurements, the resulting posteriors can be applied to all individuals with whole-genome sequencing and traits, facilitating straightforward incorporation into commonly used rare-variant testing frameworks such as burden test,^52^ SKAT,^53^ and STAAR.^54^

Combined, we present a comprehensive survey of rare variants underlying multi-omics outlier signals across the regulatory cascade. Using personal multi-omics, our Watershed model prioritized rare variants across a broad range of complex traits. These approaches further demonstrate a general and flexible framework to prioritize impactful rare variants and test for gene associations in diverse population cohorts.

### Limitations of the study

Limitations of our study include reduced signal to detect outliers impacting multiple omics measurements, which may be due to power, noise, or true biologically distinct effects. Specifically, we observed minimal sharing of outlier signals across omics data types, with relatively few multi-omics outliers. We also found that relatively few outliers in splicing and methylation survive to become mRNA and protein expression outliers; however, shared outliers in RNA and protein signals are associated with higher *Z* scores in splicing and methylation (Figure S23). These results are consistent with prior observations of regulatory mechanisms that minimize the impact of outlier levels of molecular phenotypes; yet, given the few joint outlier instances, it is still challenging to generalize these findings. Additionally, not all individuals with whole-genome sequencing had all four omics signals directly measured, and protein expression was only quantified for a limited set of genes and from plasma. Therefore, we may still be underestimating the prevalence of multi-omics outliers and missing impactful rare variants in this cohort. In this study, we employed stringent filtering steps to ascertain outliers as having consistent effects measured in two independent collections nearly 10 years apart to address potential false-positive discoveries by enriching for genetically driven outlier events. Future studies with better additional multi-omics measurements and expanded proteomic coverage will reveal more generalizable properties of outlier propagation and optimize rare-variant prioritization.

The multi-omics Watershed model was trained on all (gene, individual) pairs with omics data available, based on the hypothesis that the regulatory effects of rare variants are largely consistent across all genes and individuals. Developing Watershed models based on omics data from subsets of genes in the same biological pathways could aid ascertainment of whether certain genes and pathways are more easily perturbed by rare variants. Further, incorporation of disease cohorts and directly modeling disease status could potentially improve discovery of rare variants and causal genes underlying specific diseases. Finally, because of our gene-centric design of Watershed, we mapped rare variants to nearby genes using a 10-kb window flanking each gene. Consequently, we were not able to evaluate distal rare variants, nor were we equipped to dissect regulatory mechanisms of omics signals not directly linked to genes, such as metabolite levels. Incorporation of higher-order chromatin organization data could improve coverage of rare variants, and introduction of additional layers in Watershed to allow hierarchical modeling of omics signals would allow more flexible analysis of rare variants underlying multi-omics outliers.

## STAR★Methods

### Key resources table

**Table:** 

REAGENT or RESOURCE	SOURCE	IDENTIFIER
**Deposited data**		

Specifications of the Watershed model and resulting prioritized rare variants	This paper	Zenodo archive https://zenodo.org/record/8145312
VEP variant annotations	Cunningham et al.^55^	https://useast.ensembl.org/info/docs/tools/vep/index.html
CADD variant annotations	Rentzsch et al.^56^	https://cadd.gs.washington.edu/download
The Human Phenotype Ontology	Köhler et al.^18^	https://hpo.jax.org/app/
Open Targets	Mountjoy et al.^22^	https://www.opentargets.org/
Rare variant burden test	Cirulli et al.^20^	https://s3.amazonaws.com/helix-research- public/ukbb_exome_analysis_results/README.txt
Whole-genome sequencing and multiomic data from MESA	NHLBI TOPMed consortium	dbGaP accession dbGaP:phs001416.v3.p1 https://www.ncbi.nlm.nih.gov/projects/gap/cgi-bin/molecular.cgi?study_id=phs001416.v3.p1

**Software and algorithms**		

Probabilistic estimation of expression residuals (PEER)	Stegle et al.^57^	https://github.com/PMBio/peer/wiki/
LeafCutter	Li et al.^58^	https://github.com/davidaknowles/leafcutter
Splicing Outlier detection (SPOT)	Ferraro et al.^2^	https://github.com/BennyStrobes/SPOT/tree/master
MetaDome	Wiel et al.^17^ and this paper	https://github.com/laurensvdwiel/MetaDome-vcf-annotation Zenodo archive https://doi.org/10.5281/zenodo.817 6404
MAGMA	de Leeuw et al.^19^	https://ctg.cncr.nl/software/magma
PrediXcan	Barbeira et al.^21^	https://github.com/hakyimlab/MetaXcan
Watershed	Ferraro et al.^2^ and this paper	https://github.com/taiboli/Watershed/tree/master Zenodo archive https://doi.org/10.5281/zenodo.813 7424
Analysis scripts generated for this script	This paper	https://github.com/taiboli/MESA-Rare-Variant Zenodo archive https://doi.org/10.5281/zenodo.813 7541

### Resource availability

#### Lead contact

Further information and requests for resources should be directed to and will be fulfilled by the lead contact, Stephen Montgomery (smontgom@stanford.edu).

#### Materials availability

This study did not generate unique reagents.

### Method details

#### The Multi-Ethnic Study of Atherosclerosis (MESA)

The Multi-Ethnic Study of Atherosclerosis (MESA) is a study of the characteristics of subclinical cardiovascular disease (disease detected non-invasively before it has produced clinical signs and symptoms) and the risk factors that predict progression to clinically overt cardiovascular disease or progression of the subclinical disease.^59^ MESA researchers study a diverse, population-based sample of 6,814 asymptomatic men and women aged 45–84. Thirty-eight percent of the recruited participants are white, 28 percent African American, 22 percent Hispanic, and 12 percent Asian, predominantly of Chinese descent. Participants were recruited from six field centers across the United States: Wake Forest University, Columbia University, Johns Hopkins University, University of Minnesota, Northwestern University and University of California - Los Angeles. Participants are being followed for identification and characterization of cardiovascular disease events, including acute myocardial infarction and other forms of coronary heart disease (CHD), stroke, and congestive heart failure; for cardiovascular disease interventions; and for mortality. In addition to the six Field Centers, MESA involves a Coordinating Center, a Central Laboratory, and Central Reading Centers for Computed Tomography (CT), Magnetic Resonance Imaging (MRI), Ultrasound, and Electrocardiography (ECG). The first examination took place over two years, from July 2000 - July 2002. It was followed by five examination periods that were 17–20 months in length. Participants have been contacted every 9 to 12 months throughout the study to assess clinical morbidity and mortality.

Further, the TOPMed MESA Multi-omics Pilot successfully generated transcriptomic data by RNAseq, DNA methylation [850K CpG sites], plasma proteomics by aptamer capture (SomaLogic), and untargeted and targeted metabolomics using liquid chromatography/mass spectrometry (LC-MS from the Gerszten/Clish laboratory) in ∼1,000 multi-ethnic participants sampled at two time points, Exam 1 and Exam 5, approximately 10 years apart. These data are being used in this study. We retain only unrelated samples across all analyses. MESA whole-genome sequencing data and multi-omics data used in this study can be accessed through dbGaP accession number dbGaP:phs001416.v1.p1.

#### RNA-sequencing data generation and processing

RNA-sequencing was performed at two centers, Broad Institute of MIT and Harvard and Northwest Genomics Center (NWGC).

For Broad Institute, quantification of total RNA was accomplished using the Quant-iT RiboGreen RNA Assay Kit (Invitrogen, cat #R11490). RNA quality was assessed by RQS (RNA Quality Score) using the LabChip GX (Caliper Life Sciences). After quantification, 2 μL of a 1:1000 dilution of Ambion ERCC (External RNA Controls Consortium) RNA Spike-In Control Mix (Invitrogen, cat #4456740) was spiked into a 200 ng aliquot of each sample destined for library construction. For library construction, an automated variant of the Illumina TruSeq Stranded mRNA Sample Preparation Kit (Illumina, cat #RS-122-2103) was used where input RNA underwent two rounds of poly-A selection and was fragmented and primed for cDNA synthesis. The 3′ blunt ends of the ds cDNA were subsequently adenylated with a single ‘A’ nucleotide. This provides a complementary overhang for the ligation of adapters and prevents the cDNA fragments from ligating to each other during this ligation reaction, thereby reducing chimera formation. Molecular adapters were then ligated to the ends of the ds cDNA to serve as primers for PCR enrichment. Each adapter was a unique molecular barcode specific for each well location of the 96-well plate. After enrichment, samples were amplified using PCR and the cDNA libraries were subsequently quantified using PicoGreen and then pooled in equimolarity. The entire plate was plexed together for a maximum 94-plex. Pools were quantified using qPCR and then normalized to 2nM. Afterward, pools were denatured using 0.1 N NaOH prior to sequencing to create single-stranded DNA to be loaded onto the sequencers. Flowcell cluster amplification and sequencing were performed according to the manufacturer’s protocols using the Illumina HiSeq 4000. The runs were 101bp paired-end with an eight-base index barcode read. Raw data was analyzed using the Broad Picard Pipeline which includes de-multiplexing and data aggregation.

For NWGC, total RNA was verified using the Quant-iT RNA Assay Kit (Invitrogen, cat# Q33140) and only samples with at least 225 ng of RNA were retained. RNA Integrity Number (RIN) was estimated and verified using the Agilent 2100 Bioanalyzer (Agilent, Santa Clara, CA) (requiring minimum RIN >5 for each sample.) To control for batch to batch variation an internal control, 250ng of K-562 total RNA (Thermo Fisher Scientific, cat# AM7832), was added to each 96-well plate processed. Plate-to-plate expression correlation of K-562 was typically >0.99. For library construction, Total RNA was normalized to 5ng/ul in a total volume of 50ul on the PerkinElmer Janus Workstation (PerkinElmer, Hopkington, MA). Poly-A selection and cDNA synthesis were performed using the TruSeq Stranded mRNA kit as outlined by the manufacturer (Illumina, cat#RS-122-2103). Total RNA was subject to two rounds of poly-A selection through sequential binding of poly-A RNA to oligo d(T) beads and washing away of unbound RNA. Purified mRNA was then eluted from the beads, fragmented and randomly primed for first strand synthesis using the SuperScript III reverse transcriptase (Invitrogen, cat#18080085). The original RNA template was degraded and double stranded cDNA was made using the first strand of cDNA as a template. The resulting cDNA was purified using AMPure XP beads (Beckman Coulter, A63882). Double-stranded cDNA proceeded through a series of shotgun library steps using the TruSeq Stranded mRNA kit, as outlined by the manufacturer. Library molecules are adenylated (A-tailing) to accommodate the T overhang of the Illumina Truseq adapters. Full length adapters were then ligated to the cDNA fragments, followed by an AMPure XP cleanup to remove unligated adapters. A dual indexing strategy was adopted to avoid index hopping and to uniquely identify each library. Adapter ligated ds cDNA molecules were amplified by 13 cycles of PCR and subjected to a final 1X AMPure XP cleanup to remove carry over primers. All library preparation steps were carried out on the PerkinElmer Sciclone NGSx Workstation to reduce batch to batch variability and to increase sample throughput. Final RNASeq libraries were quantified using the Quant-it dsDNA High Sensitivity assay. Library insert size distribution was checked using the DNA1000 assay on the Agilent 2100 Bioanalyzer. Samples where adapter dimers constituted more than 4% of the electopherogram area were failed prior to sequencing. Technical controls (K562) were compared to expected results to ensure that batch to batch variability was minimized. Successful libraries were normalized to 10nM for submission to sequencing. Ninety-six normalized and indexed libraries were pooled together and denatured before cluster generation on a cBot. The 96-plex pools were loaded on eight lanes of a flow cell and sequenced on a HiSeq4000 using illumina’s HiSeq 4000 reagents kit (cat# FC-410-1001,1002). For cluster generation, every step is controlled by cBot. When cluster generation is complete, the clustered patterned flow cells are then sequenced with sequencing software HCS (HiSeq Control Software v3.4.0.38). The runs are monitored for %Q30 bases using the Sequencing Analysis Viewer. Using RTA 2 (Real Time Analysis 2 v2.7.7), the base calls were de-multiplexed.

For data analysis, the scripts and reference annotations used to quantify transcripts mapping to each gene are available and described here: https://github.com/broadinstitute/gtex-pipeline/blob/master/TOPMed_RNAseq_pipeline.md. Briefly, RNA-seq reads were aligned using STAR^60^ to the GRCh38 reference genome, and gene quantification and quality control was done using RNA-SeQC,^61^ resulting in read counts and number of transcripts per million mapped reads (TPM). As described in chapter 3, we log2-transformed the expression values (log_2_(TPM +2)), using the GENCODE v30 gene annotation, available at the above URL. We subsetted to autosomal lincRNA and protein-coding genes and restricted to genes with at least 6 reads and TPM >0.1 in at least 20% of individuals.

#### DNA methylation data processing

DNA methylation measurements were obtained from whole blood using the Illumina EPIC chip. Initial quality control and normalization was performed using the meffil R package^62^ for functional normalization. Briefly, quality control included assessing sample swaps, of which two were identified and resolved, sample call rate, sex detection mismatches, and genotype concordance. We exclude samples where >5% of CpG sites had a detection p value >0.01 and those that were visible outliers based on their ratio of methylated to unmethylated signal. We also remove three samples whose genotypes did not match the SNP probes included on the microarray (concordance threshold = 0.8). After removing samples that failed QC, we apply functional normalization,^63^ which extends the idea of quantile normalization to adjust for unwanted technical variation via control probe PCs. We used 10-fold cross validation to determine the number of control PCs to include based on the residual variance after fitting 20 PCs, and decided to include 11, based on decreases in the residuals. We apply some additional filtering: (1) Remove individual-site instance if detection p value >0.01, (2) Exclude probes if > 10% of values are missing, (3) Several filters based on mapping issues, non-CpG targeting, or polymorphisms as described in,^64^ with suggested masking and variant annotations for this array available here: http://zwdzwd.github.io/InfiniumAnnotation. Importantly, we removed sites if there exists a common SNV that overlap the measurement probe region, and we also removed individual-site pairs if the individual carries a rare variant in the probe’s target region.

#### Plasma proteome data processing

Proteome measurements were obtained via the SOMAscan HTS Assay 1.3K - Plasma, which is a highly multiplexed, aptamer-based assay,^65^ with assessment of this array in particular described in.^65^^,^^66^ SomaLogic suggests several normalization steps, starting with the observed Relative Fluorescence Intensities (RFUs) which are compared against reference values and scaled accordingly (denoted by SomaLogic as Hybridization Control Normalization or Hyb). They also apply Median Signal Normalization (Hyb.MedNorm), which is an intraplate normalization procedure to remove sample-to-sample differences that may be due to overall protein concentration or experimental variation. Then there is a calibration step (Hyb.MedNorm.Cal), based on the levels of each analyte within calibrator replicates. These steps are described in further detail in.^66^

#### eOutlier calling

To identify expression outliers, we take the log-transformed TPM values within each exam for autosomal lincRNA and protein-coding genes and identify 30 hidden factors associated with technical variation via PEER.^57^ We then run a linear regression model with the log-transformed TPM values as the outcome variable and the 30 hidden factors, top 11 genotype PCs, genotype of the strongest *cis*-eQTL per gene, age, and sex as predictors.

We compute the model residuals using the lm() function in R. We scale the residuals from that regression to generate Z-scores within each exam. We then require either the outlier or non-outlier signal at a given threshold to be seen in both exams for all downstream analyses, except for assessing replication across exams, where we apply the thresholds in each exam separately. We remove individuals that have a number of outlier genes (|Z| > 3 in both exams) greater than 1.5∗IQR based on the distribution of eOutlier burden across all individuals.

#### mOutlier calling

To identify methylation outliers, we first transform the normalized beta values to m-values^67^:

$$m=\log_{2}\left({beta}_{i}\;/\;\left(1-{beta}_{i}\right)\right)$$

From there, we apply the same correction approach as described above, though hidden factors via PEER^57^ were learned on inverse normalized beta values from a random subset of 100,000 CpG sites. We correct for 30 PEER factors, the top 11 genotype PCs, genotype of the strongest *cis*-mQTL per gene (with FDR <0.25), age, and sex, and again scale the residuals to generate Z-scores per CpG site. We then filter out sites with any common SNVs overlapping the measurement probe, including the CpG site, and individual-site instances if the individual carries a rare variant within the probe region, and retain only autosomal sites. We remove individuals that have a number of outlier CpG sites (|Z| > 3 in both exams) greater than 1.5∗IQR based on the distribution of mOutlier burden across all individuals. To calculate gene-level methylation Z-scores, we take the median *Z* score across all measured CpG sites within 1.5kb upstream of a gene’s TSS, restricting to protein-coding and lincRNA genes, as in the expression analyses.

#### sOutlier calling

We applied the SPOT (SPlicing Outlier deTection) framework to detect splicing outliers similar to previous work.^2^ Briefly, we performed intron clustering by adapting a LeafCutter pipeline^58^ from STAR^60^ -aligned junction reads. We applied custom filtering to remove exon-exon junctions with low expression while retaining rare junctions by excluding junctions where no sample has ≥ 15 reads, and further excluded exon-exon junctions with less than 40% of samples with more than three reads. We then applied the SPOT pipeline to first fit a Dirichlet-Multinomial distribution to counts spanning alternatively spliced exon-exon junctions for each gene, based on which we then identified individuals with significant deviation away from the population mean based on Mahalanobis distance (MD) metric. To avoid biases caused by dimensionality where clusters with larger number of exon-exon junctions show different MD distribution compared to smaller clusters, we computed the empirical p values for each individual in each cluster by comparing against 10,000 random samples from the fitted Dirichlet-Multinomial distribution. To map empirical p values from intron clusters to genes, we took the minimum p value (*p*_m_) across all c clusters within the span of each gene and computed the conservative estimate of the probability of observing pm across c independently drawn uniform distributions as

$$P_{gene}=1-{\left(1-p_{m}\right)}^{c}$$

Finally, we converted p values to Z-scores assuming a normal distribution.

#### pOutlier calling

For protein outliers, we natural log transform the normalized fluorescence values, before proceeding with the same correction procedure, where we identify 30 hidden factors via PEER^57^ and correct for those in addition to the top 11 genotype PCs, genotype of the strongest *cis*-pQTL per gene (with FDR <0.25), age, and sex, before scaling the residuals within each exam to generate Z-scores. We remove individuals that have a number of outlier proteins (|Z| > 3 in both exams) greater than 1.5∗IQR based on the distribution of pOutlier burden across all individuals.

#### Outlier sharing analysis

We assessed consistency of Z-scores by Pearson correlation across individuals for omic signals measured at two different time points and determined significance using the *t*-statistic, where Bonferroni correction was used to control for Type I error. We defined outlier (gene, individual) pairs in each omics data to have |Z| > 3 in both time points (joint outliers), where we also assessed outlier sharing across time points and across the regulatory cascade at varying threshold of |Z| (between 2 and 10). For joint outliers in each data type, we calculated mean Z-scores across exams, and assessed outlier sharing by taking a set of (gene, individual) pairs with |Z| > 3 in one signal and calculated proportion of these (gene, individual) pairs in each of the other signals with a relaxed threshold |Z| > 2, for the set of genes and individuals with both data types measured. For expression, methylation, and protein levels, we also assessed outliers with Z < −3 (under outliers) and those with Z > 3 (over outliers) separately.

#### Variant calling and annotations

Whole genome sequencing was generated as described in.^68^ We restrict our analysis to single nucleotide variants and small insertions and deletions that appear at a less than 1% frequency across MESA as well as < 1% across the entire gnomAD dataset^69^ and <1% in all relevant gnomAD sub-populations, including non-Finnish European, African, East Asian, and American. We annotated the VCF using Ensembl VEP (version 103^55^). CADD^56^ scores were extracted from a pre-compiled annotation file (https://cadd.gs.washington.edu/download) using variant scores from the hg38 genome build.

#### Rare variant enrichment analysis

Enrichments were calculated by restricting SNVs and indels to those that occur at less than 1% frequency across MESA and for those found in gnomAD,^69^ also at less than 1% frequency across all of gnomAD, as well as relevant sub-populations (see above). For eOutliers and pOutliers, we intersect variants with the gene body +\- 10kb on either end of the gene, based on gencode v26 annotations: https://www.gencodegenes.org/human/release_26.html. For mOutliers, we intersect variants with varying window sizes around the CpG site, using bedtools.^70^ After intersecting variants, we convert to a binary signal, with 1 indicating at least one rare variant was found in the region for that individual and 0 indicating no rare variants. For additional annotations, we subset the full set of rare variants to those with the given annotation, as determined via VEP,^55^ release 103, and CADD,^56^ version 1.6. Additionally, we assessed whether each variant lies within protein meta-domains as identified by MetaDome^17^ after lifting over all variants to hg19.

We calculated enrichment as the ratio between outlier and control individuals for the proportion of (gene, individuals) with rare variants within 10kb window of the gene body, restricting to the same set of genes with outlier measurements as before.^2^ The relative risk and confidence intervals were estimated from two-way contingency tables summarizing (gene, individual) pairs for their outlier status (outlier vs. control) and whether they contain a rare variant in each annotation, using the epitools package in R. Significance of enrichment was calculated by one-sided t-test against null (relative risk of 1). We define non-outliers as those with |Z| < 1 in both exams. To compare two enrichment tests, we estimated *t*-Value by the difference in the log (relative risk) divided by the overall standard error of the difference, which was converted to p value with degree of freedom equal to the number of total outlier and control instances used in the relative risk calculation minus 2. We compared gene-level enrichments for all signals, which required us to summarize Z-scores for all probes and splicing clusters mapped to the same gene for methylation and splicing outlier signals.

#### Effects of ancestry on rare variant enrichment

To assess whether the observed enrichments were impacted by differences in genetic ancestry between outlier and non-outlier individuals within each set, even after correcting for the top 11 genotype principal components (PCs) before identifying eOutliers, mOutliers, and pOutliers, we calculated the correlation between individual outlier burden (the number of outliers per data type identified for a single individual) and loadings on genotype PC values for all outlier types, across different thresholds for the definition of outliers. We further replicated rare variant enrichment tests by matching each outlier individual to a non-outlier individual based on closest Euclidean distance defined by the top 11 genotype PC values and compared the resulting relative risk estimates with those from corresponding tests retaining all non-outlier individuals. We selected a control individual for each outlier by taking the individual from the full set of controls (|exam 1 *Z* score| < 1 and |exam 5 *Z* score| < 1) with the lowest Euclidean distance to the outlier individual based on the top 11 genotype PC values. Distances were calculated using the philentropy R package.

#### Replication with rare variants in GTEx

We subset rare variants seen in GTEx v8 to those associated to multi-tissue expression outlier effects. We assessed the subset of the rare SNVs and indels observed in GTEx that were also seen in any individuals in MESA and calculate the proportion that also led to observable outlier effects at the methylation, expression, and protein level. We assessed significance of this overlap based on permutations, where within MESA, we permuted Z-scores, keeping measurements together across time points, within each gene across individuals and assess the number of times a GTEx eOutlier variant is associated with an expression change in MESA. The empirical p value was calculated based on 100 permutations.

To assess enrichment of different types of variants in the set of replicating variants as compared to the remaining variants, for each annotation, we created a contingency table where the rows indicate whether or not the variant effect from GTEx was observed in MESA and the columns contain the number of variants with and without the given annotation. We then calculated a relative risk of a variant associated with a replicating effect having a given predicted effect, i.e., annotation again using the epitools R package to estimate the relative risk and confidence intervals.

#### Overview of the multi-omics Watershed model

To integrate genomic annotation with outlier signals to prioritize rare variants with large effects across the regulatory cascade, we extended our Bayesian hierarchical model, Watershed, which consists of a layer of genomic annotation variables (G), a fully connected layer (Z) of latent regulatory variables for each of the four omic signals (mRNA expression, methylation, splicing, and protein expression), and a layer of variables (E) representing the observed outlier status of each omics data type. We used as input p values for each of the four signals and for all (gene, individual) pairs with at least two signals measured in MESA, along with a set of 77 binary and continuous genomic annotations aggregated across all rare variants nearby each gene. These annotations, listed in Table S1, served as priors in the Watershed model and as input to the genomic annotation model (GAM) using logistic regression. We curated a comprehensive set of annotations and induced l_2_ sparsity constraints on both Watershed and GAM to allow our models to learn the most informative set of features. Watershed was then trained to learn edge weights connecting each variable and estimate posterior probability of each rare variant leading to outlier levels of nearby gene for each of the four signals, given genomic annotations and observed expression levels P(E|G, Z). As evaluation, we identified pairs of individuals with the same set of rare variants nearby the same gene (N2 pair), and asked the Watershed model to predict the regulatory status of one individual in the pair based on genomic annotations of rare variants and observed outlier status of each omic signal in the other individual. We benchmarked the performance of multi-omics Watershed model against GAM and Bayesian models based on single outlier signals at a time (RIVER), at the same p value threshold for defining outliers (p < 0.05). We repeated the same analysis when training the model on individuals of European ancestry and evaluating its performance on individuals from other ancestries to assess its cross-population portability.

#### Details of Watershed Implementation

Watershed was designed to model instances of (gene, individual) pairs given the functional annotations of nearby rare variants and observed outlier status of gene measurements. The multi-omics Watershed model requires two kinds of input variables:

1. A set of genomic annotation variables for each rare variant: We curated a list of N = 73 annotations for each variant, consisting of variant effect predictor (VEP) consequences, regulatory element annotations, conservation scores, and other genomic and epigenomic features from other models such as CADD and ENCODE, detailed in Table S1. For each (gene, individual) pair, we aggregated each annotation across all rare variants within the 10kb window of the gene. Finally, we performed mean centering and scaling for each genomic annotation variable as input to the Watershed and GAM models.

2. A set of categorical variables representing the observed outlier status of the gene: Based on the estimated p values in each signal (mRNA expression, methylation, splicing, and protein expression), we binarized our (gene, individual) pairs as outliers in each dimension at a p value threshold of 0.05 (--pvalue_threshold = 0.05 as input to the model). In practice, this translates roughly to the top 2% of Z-scores in each signal (at a *Z* score cutoff of around 2), which we also used for evaluation of the model (--pvalue_fraction = 0.02). We explicitly modeled over- and under-outliers for RNA, methylation, and protein levels.

To enrich for outlier signals with genetic effects, we only included genes with consistent Z-scores across the two visits by removing (gene, individual) pairs where one visit has |Z| ≥3 and the other has |Z| ≤ 1. We used median Z-scores across visits as input to Watershed. We also removed individuals who have significantly higher number of outlier measurements (“global outliers”), defined as those having more than Q3 + 1.5∗IQR outliers in each signal (Q3 represents 75th percentile rank value and IQR represents interquartile range). Further, to model relationships between omic signals, we only kept (gene, individual) pairs with at least two types of omic measurements (out of four), and filtered out genes which do not have any outlier individual in at least two measurements.

We applied Watershed exact inference optimization routine which is tractable for K = 4 outlier signals. To evaluate the performance of the multi-omics Watershed model, we took pairs of individuals with the same set of rare variants nearby the same gene (“N2 pairs”), who were not included in the training of the model, to assess the ability of the model to predict regulatory status of the second individual based on genomic annotations and observed outlier status of the first individual in each dimension, using area under the precision-recall curve as a metric. In total, we had 596,288 instances of (gene, individual) pairs from 932 unique individuals, of which we had 60,724 N2 pair individuals for evaluation of the model.

To estimate Watershed posterior probabilities, we applied the trained multi-omics Watershed model and calculated P(Z |G, E) for all rare variants from all (gene, individual) pairs in MESA (description of variables in Figure S13A). We scored posterior probability for a total of 30 million (gene, individual, rare variant) triplets. By varying Watershed posterior threshold from 0.2 to 0.8, we identified between 19,589 and 199,790 variants with evidence of driving at least one outlier signal. Importantly, Watershed can model missing outlier measurements such that each variant has posterior calculated in all four signals. We assigned a final posterior estimate for each rare variant by taking the maximum across all individuals with the variant. We provided the list of prioritized variants at posterior threshold 0.8 in each outlier signal in Table S2.

Of note, we also trained a Watershed model using signals obtained from RNA-seq alone (mRNA expression and splicing) to assess potential improvements of the multi-omics model over our previous work based on single data modality. The expression + splicing (E + S) model showed similar performance when evaluated on the same set of N2 pair individuals. However, it prioritized less than 1/4 of variants identified by the multi-omics model. Another unique advantage of the multi-omics model is that we can estimate a variant’s effect as long as we have data from at least one out of the four outlier signals. In other words, multi-omics Watershed can be applied to any new cohorts with limited data, and estimate the effects of variants on all four omic signals.

#### GAM and RIVER

We followed previous procedures in training genomic annotation models (GAM) and RIVER models.^1^ Briefly, for GAM, we applied a logistic regression model using all genomic annotations as input features and l2 regularization to promote sparsity. We used as the response variable the binarized outlier status (a p value threshold of 0.05) for each signal. For RIVER, we ran separate Watershed models with one outlier signal in each because it is a special case of Watershed.

#### Cross-population comparison of Watershed performance

Based on the seven super populations present in the Human Genome Diversity reference panel,^14^ we applied RFmix^71^ to estimate genetic ancestry for each individual in MESA. We assigned individuals to ancestries if they have a probability >0.75 of belonging to the group, with the exception of Hispanic population (Native Americans) where we used a threshold of 0.5 to include more people. As a result, we identified 426 Europeans, 270 Africans, 107 East Asians, and 54 Hispanic individuals.

To assess cross-population portability of multi-omics Watershed, we constructed training data from 147,569 (gene, individual) pairs, following the same inclusion and exclusion criteria as the main model, where all individuals are of European ancestry. We then constructed evaluation data using N2 pairs where both individuals come from the same population. We applied the Watershed model trained from European individuals and tested its performance separately on N2 pairs from other populations in each omic signal, where we excluded populations with less than ten N2 pairs in each signal for evaluation. To compare statistical difference, we designed a bootstrapping procedure to subsample half of N2 pairs in each test set 100 times and computed the distribution of area under the precision-recall curve across bootstrapped samples. Because of sample size differences and the different total variant burden in each population, our power to prioritize rare variants depended on ancestry. In total, we identified most variants from individuals with African ancestry (16K at threshold of 0.5) followed by those with European ancestry (13K). East Asians and Hsipanic/Latinx populations contributed to 2.7K and 1.4K prioritized variants.

#### Correlation of Watershed posteriors with GWAS effect size

Because Watershed predicts functional impact or rare variants across the regulatory cascade, we reasoned that Watershed prioritized variants affecting essential genes related to a trait should have a high functional impact on the trait. To systematically test this hypothesis, we identified several polygenic traits with summary statistics and compared the distribution of estimated effect size with variants prioritized by Watershed at different posterior thresholds.

Specifically, we considered the following traits：

- Height: We obtained summary statistics on rank-normalized standing height from UK Biobank release 2 with 361,194 individuals of both sexes. This data includes imputed genotypes from HRC plus UK10K & 1000 Genomes reference panels as released by UK Biobank in March 2018.
- Rheumatoid arthritis: We obtained summary statistics from a *trans*-ethnic meta-analysis with 19,234 cases and 61,565 controls.^72^.
- COVID-19 severity: We obtained summary statistics comparing severe positive cases with non-severe positive cases based on 1,244 cases and 16,413 controls from UK Biobank released in May 2021.
- Alzheimer’s Disease (AD): We obtained summary statistics from a recent meta-analysis which includes 90,338 cases and 1,036,225 controls.^73^.
- Schizophrenia: We obtained summary statistics from the most recent public release from the Psychiatric Genomics Consortium (wave 3) consisting of 67,390 cases and 94,015 controls.^74^.

For each trait, we first lifted the summary statistics to GRCh38 genome assembly and intersected with all rare variants present in MESA. This step resulted in varying amount of variants left because of differences in sequencing platform and imputation strategy from different studies. In total, we had a range of thousands (schizophrenia) to hundreds of thousands (COVID-19 severity) rare variants with both effect size estimation and posterior probabilities leading to outlier levels in four omic signals from multi-omics Watershed.

We then applied percentile normalization on variant effect sizes for all such rare variants (background, all rare variants regardless of Watershed posterior). The distribution of normalized effect size was then compared across rare variants prioritized by Watershed at varying thresholds (0.5 or 0.9), where we focused on variants nearby genes with known evidence of being causal for each trait through the Human Phenotype Ontology (HPO^18^) or Open Targets.^22^ Given the differences in posterior distribution in each omics data type, we also considered same number of top N variants as prioritized by Watershed for each data type in isolation (N = 10 and 100) and compared their effect size distribution against the background. Additionally, because Watershed can leverage data from all outlier signals and make posterior predictions even for unobserved omic measurements, we repeated this analysis after subsetting to variants mapped to directly measured genes in each signal.

#### Gene-based test for association with height using Watershed posteriors

Among the 1,319 individuals with multi-omics measurements in MESA, we computed posterior probabilities for each rare variant leading to outlier RNA expression, methylation, splicing, or protein expression in nearby genes. We reasoned that these posteriors can directly reflect the functional impact of these variants on traits, particularly if they map to genes with strong evidence of association with the trait. We also noted that once trained, these posteriors can be applied to a much larger set of individuals with whole genome sequencing data for association testing as long as they share those rare variants identified in the original training cohort; therefore, Watershed posteriors can be used in a general framework as weights for gene association testing, most appealingly in biobank scale data which do not need to have multi-omics measurements.

As a proof of concept for this workflow, we collected standing height from 4,559 individuals with whole genome sequencing and computed residual height after regressing out age, sex, self-reported race, clinical center, and top 10 genotype PCs. We defined individuals with residual |Z| > 2 as outlier individuals, and those with residual |Z| < 0.2 as controls, resulting in 182 outlier individuals and 680 control individuals. We next collapsed all rare variants located within 10kb of the gene body for each gene, and applied Wilcoxon rank-sum tests to assess the difference in distribution of posteriors in outlier and control individuals. This gene-level test is similar to the burden test framework but uses Watershed posteriors as weights and incorporates many more non-coding variants. For each gene, we compared Watershed posterior distribution for all rare variants in outlier individuals versus controls using a Wilcoxon rank-sum test and controlled for multiple testing by the Benjamini-Hochberg procedure. We applied this analysis using posteriors from each signal and a combined posterior summarizing the largest effects across all four signals.

We compared our test with three well-established gene prioritization methods:

1. MAGMA: we applied MAGMA gene analysis^19^ using the same GWAS summary statistics on height from UK Biobank and the top SNP model to derive gene-level p values.
2. PrediXcan: we applied PrediXcan using the same GWAS summary statistics on height from UK Biobank (S-PrediXcan) and MASHR-based eQTL models trained across 49 tissues in GTEx.^21^^,^^75^^,^^76^ We chose the minimum p value across tissues to obtain gene-level *p* values.
3. Burden test: we obtained precalculated burden test results from Helix^20^ which is also based on UK Biobank data. We used BOLT-LMM p values in our comparison.

### Quantification and statistical analysis

Statistical analyses and visualization were performed using R version 4.0.3. Significance was determined at p < 0.05 after Bonferroni correction. Description of statistical tests performed and sample size (number of individuals, genes, rare variants, and instances of [variant, gene, individual] pairs) can be found in corresponding sections of the main text, and details of statistical analyses were included in STAR Methods, Supplemental Information, and figure legends where applicable.

## Data Availability

Raw whole-genome sequencing and multi-omics data have been deposited at dbGaP with accession numbers listed in the key resources table. All original code and data supporting analyses presented in this paper has been deposited on Github and Zenodo and is publicly available as of the date of publication. DOIs are listed in the key resources table. List of prioritized variants have been provided as a supplemental table and deposited at Zenodo. Any additional information required to reanalyze the data reported in this paper is available from the lead contact upon request.

## Supplemental information

Document S1. Figures S1–S23

Table S1. Genomic annotations in the multi-omics Watershed model, related to Figure 4

Table S2. Prioritized rare variants by the multi-omics Watershed model, related to Figure 5

Document S2. Article plus supplemental information
