# Supplementary material for: The functional impact of rare variation across the regulatory cascade
Source: Cell Genom. 2023 Sep 6;3(10):100401. doi: 10.1016/j.xgen.2023.100401 (PMC10589633; doi:10.1016/j.xgen.2023.100401)
Supplement: Document S1. Figures S1–S23 [file mmc1.pdf]

Cell Genomics, Volume *3*

Supplemental information

The functional impact of rare variation

across the regulatory cascade

Taibo Li, Nicole Ferraro, Benjamin J. Strober, Francois Aguet, Silva Kasela, Marios Arvanitis, Bohan Ni, Laurens Wiel, Elliot Hershberg, Kristin Ardlie, Dan E. Arking, Rebecca L. Beer, Jennifer Brody, Thomas W. Blackwell, Clary Clish, Stacey Gabriel, Robert Gerszten, Xiuqing Guo, Namrata Gupta, W. Craig Johnson, Tuuli Lappalainen, Henry J. Lin, Yongmei Liu, Deborah A. Nickerson, George Papanicolaou, Jonathan K. Pritchard, Pankaj Qasba, Ali Shojaie, Josh Smith, Nona Sotoodehnia, Kent D. Taylor, Russell P. Tracy, David Van Den Berg, Matthew T. Wheeler, Stephen S. Rich, Jerome I. Rotter, Alexis Battle, and Stephen B. Montgomery

Supplemental Figures

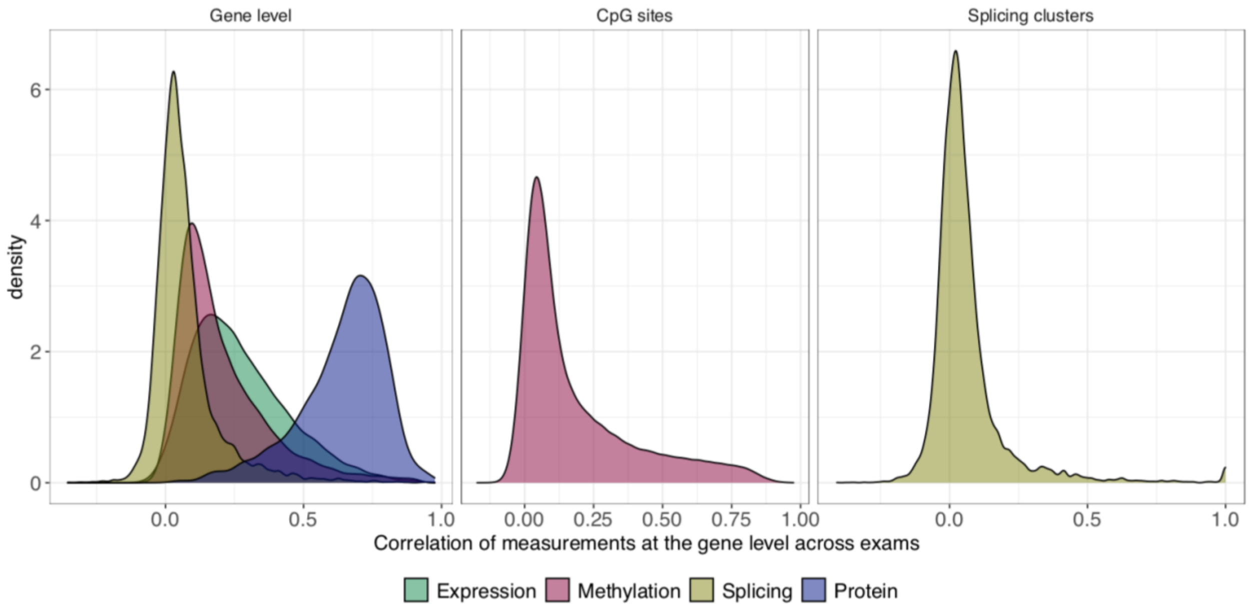

Figure S1. Correlation of corrected measurements over time, related to Figure 1. The distribution of Pearson correlation coefficients across expression Z-scores (green), gene-level methylation Z-scores (red), gene-level splicing Z-scores (gold), and protein Z-scores (blue) on the left, with the correlation across Z-scores per CpG sites in the center and across splicing clusters on the right.

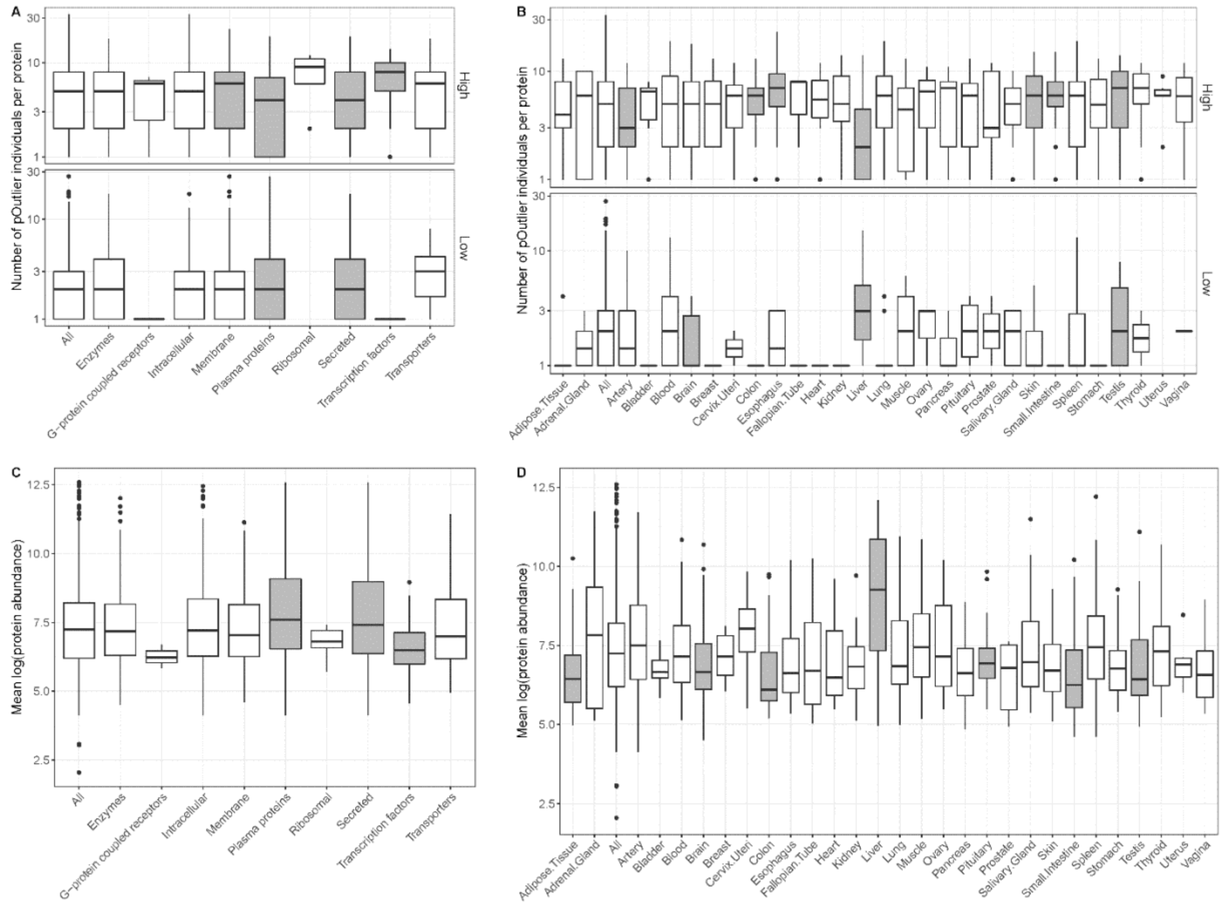

Figure S2. Distribution of pOutlier burden across proteins by class and tissue, related to Figure 1. (A) The distribution of number of pOutlier individuals across proteins within different classes, as annotated by the Human Protein Atlas, split by the direction of effect with high abundance pOutlier burden on the top and low abundance pOutlier burden on the bottom. **(B)** The mean log(protein abundance) across all individuals for proteins annotated to different classes. **(C)** The distribution of number of pOutlier individuals across proteins annotated as either enhanced or enriched for given GTEx tissues, split by the direction of effect with high abundance pOutlier burden on the top and low abundance pOutlier burden on the bottom. **(D)** The mean log(protein abundance) across all individuals for proteins annotated to different tissues. For all panels, the shaded grey boxes indicate a significant difference between the values for that class or tissue and the set of all proteins, as determined by a two-sided Wilcoxon rank sum test with $p < 0.05$.

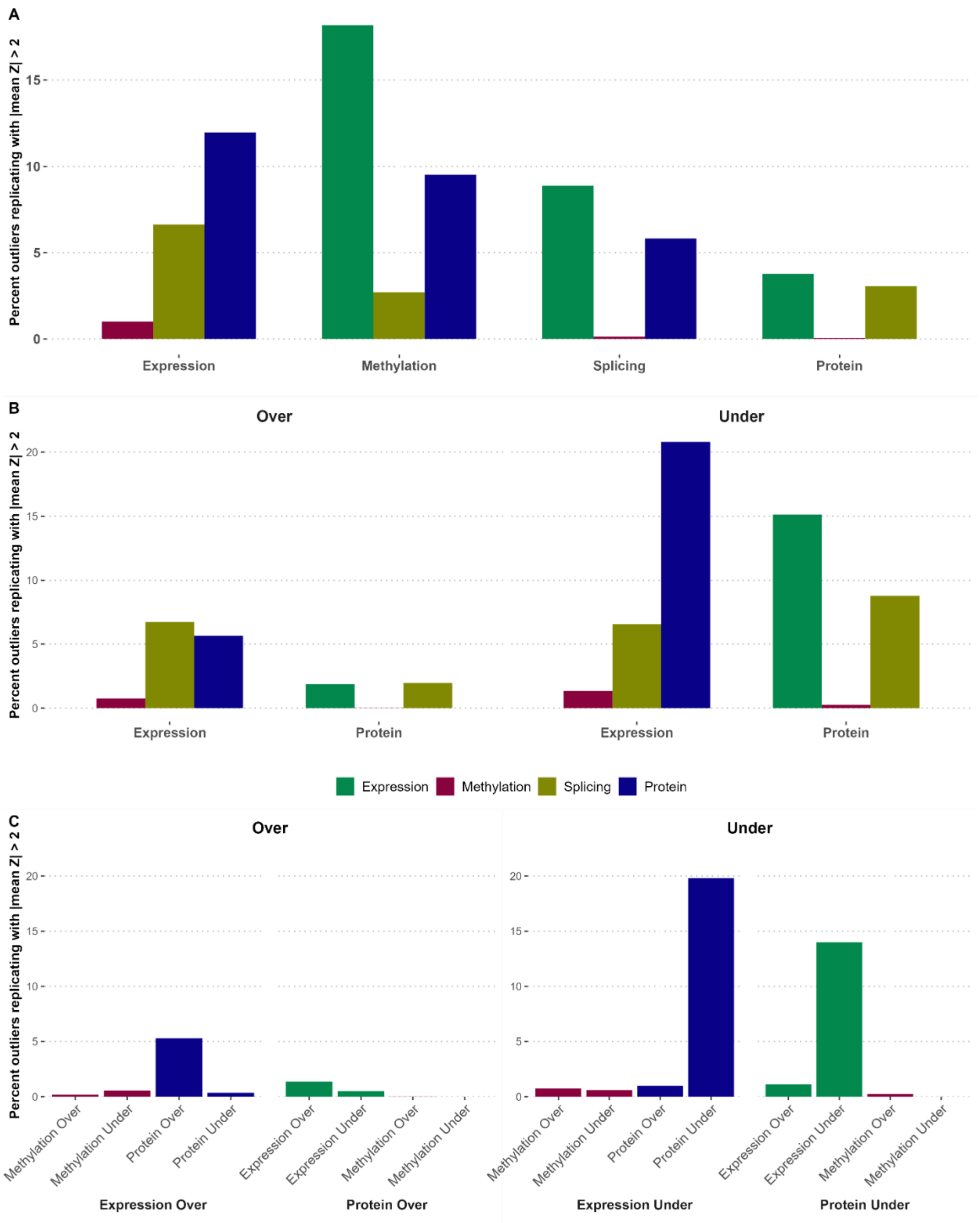

Figure S3. Replication of outliers across data types, related to Figure 2. (A) The percent of outliers (y-axis) identified within each data type (x-axis) that are also seen across each other data type at a threshold of |mean Z| > 2 across exams, considering the set of genes and

individuals measured in both. **(B)** The percent of eOutliers and pOutliers (y-axis) identified within each data type (x-axis) that are also seen across each other data type at a threshold of |mean Z| > 2 across exams, considering the set of genes and individuals measured in both, split by the direction of the outlier effect in the discovery data type (expression or protein levels, respectively). **(C)** The percent of eOutliers and pOutliers that replicate in other data types, split by both the direction of the outlier effect in the discovery data type (expression or protein levels) and the direction of replicating data type (shown by colors)

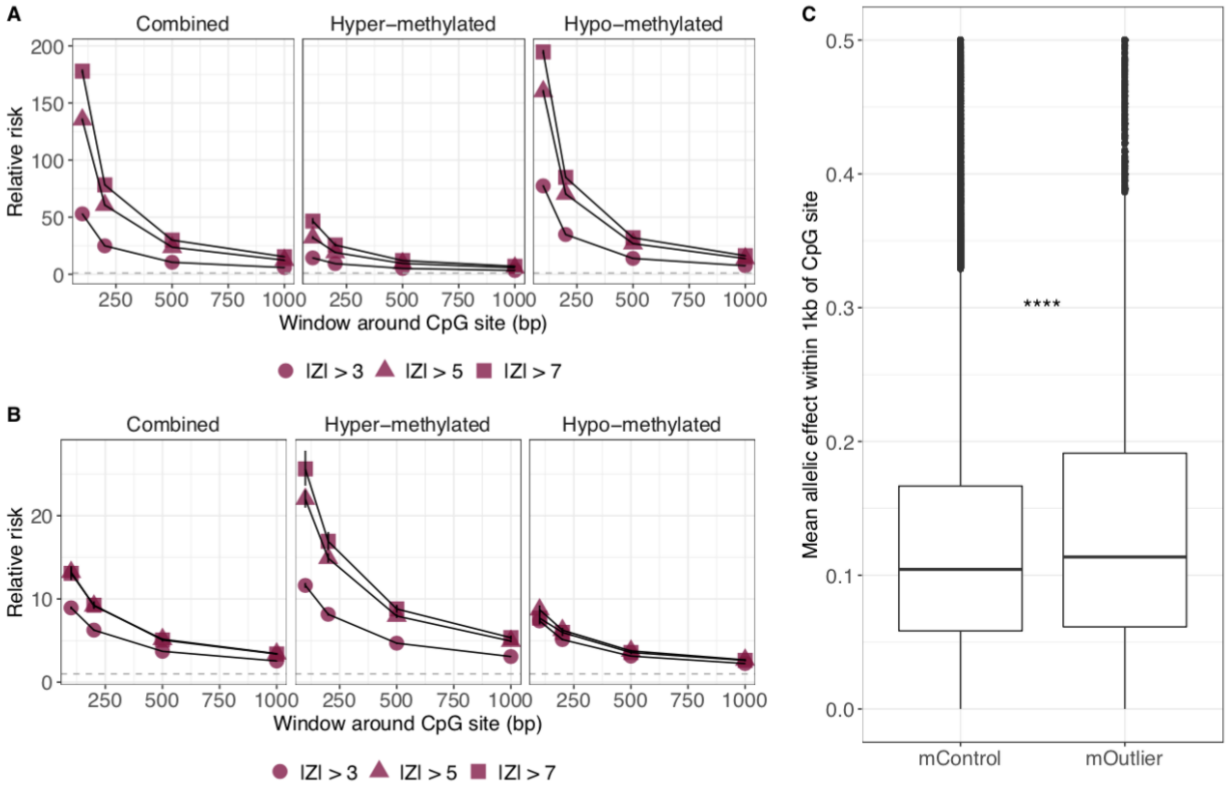

Figure S4. Enrichment of rare variants nearby CpG-level mOutliers, related to Figure 3. **(A)** The relative risk of carrying a nearby rare variant within varying distances from the outlier CpG site (x-axis) across thresholds (shapes) for CpG-level mOutliers identified across both exams (left), as well as the subset that show hyper-methylation (center) and hypo-methylation (right). Any probes that overlap common SNVs have been filtered out, as well as individual-probe instances if a rare variant overlaps the measurement probe as well. **(B)** The relative risk of carrying a nearby rare variant within varying distances from the outlier CpG site (x-axis) across thresholds (shapes) for CpG-level mOutliers identified across both exams (left), as well as the subset that show hyper-methylation (center) and hypo-methylation (right). Any probes that overlap common SNVs have been filtered out, as well as individual-probe instances if a rare variant overlaps the measurement probe and/or the CpG site as well. **(C)** For the set of CpG-level mOutliers assessed in (A), the mean allelic effect measured for any SNVs within a 1kb window around mOutliers as well as control individuals for the same set of sites. $p < 2.2e\text{-}16$ based on a one-sided Wilcoxon rank sum test.

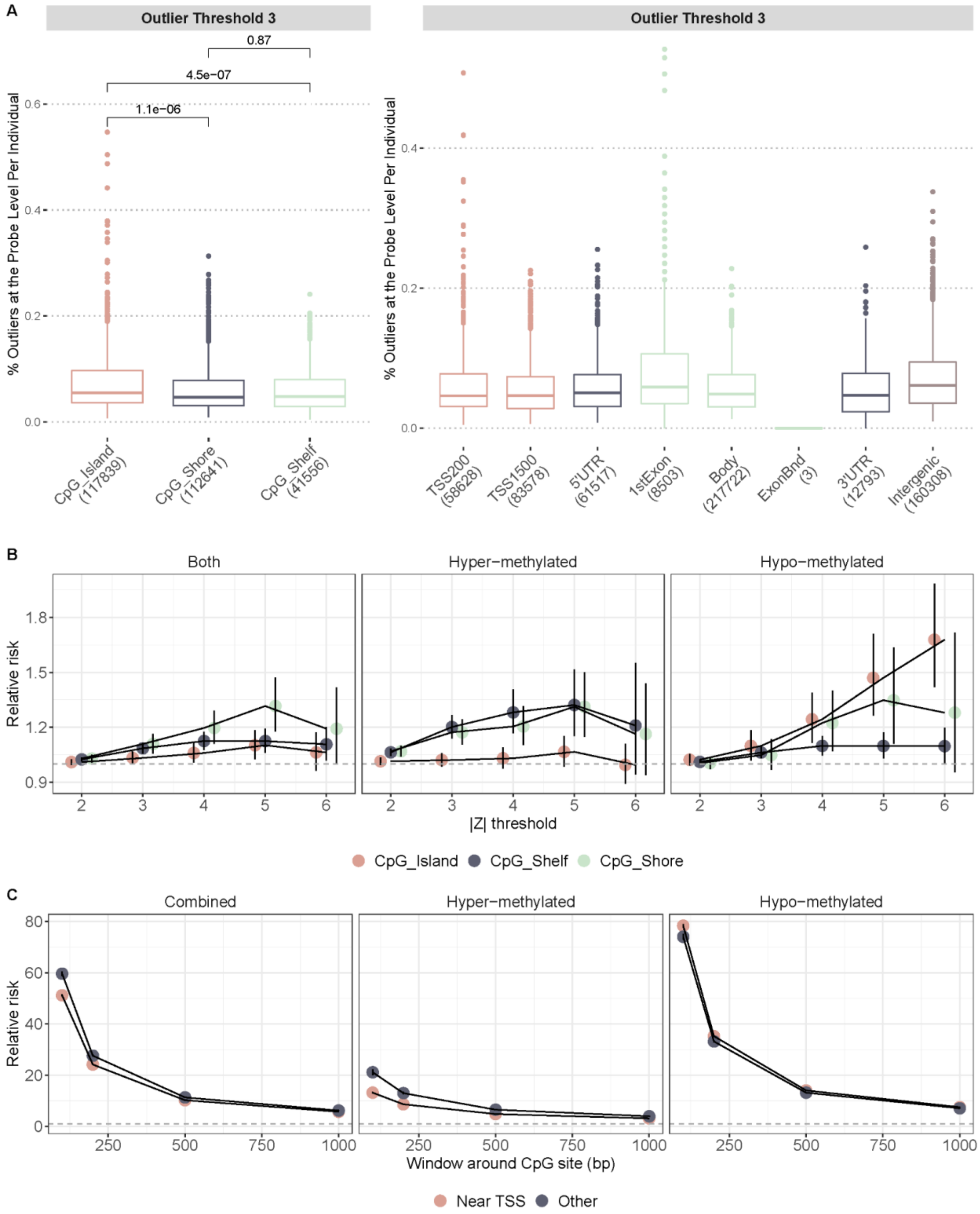

Figure S5. mOutliers at different genomic locations, related to Figure 3. (A) Distribution of the percentage of methylation probes which are outliers (at |Z| > 3) for each individual, grouped by the genomic locations of each type of probes (x-axis, numbers in parentheses indicate total

number of probes in each category), relative to CpG islands (left) and promoters (right). Probe annotations are obtained from Infinium MethylationEPIC Manifest by Illumina. **(B)** The relative risk of carrying a nearby rare variant with varying outlier threshold (x-axis) across methylation probes in and around CpG islands (colors) for mOutliers identified across both exams (left), as well as the subset that show hyper-methylation (center) and hypo-methylation (right). **(C)** The relative risk of carrying a nearby rare variant within varying distances from the outlier CpG site (x-axis) across methylation probes near (within 1.5kb) and away from TSS (shapes) for CpG-level mOutliers identified across both exams (left), as well as the subset that show hyper-methylation (center) and hypo-methylation (right).

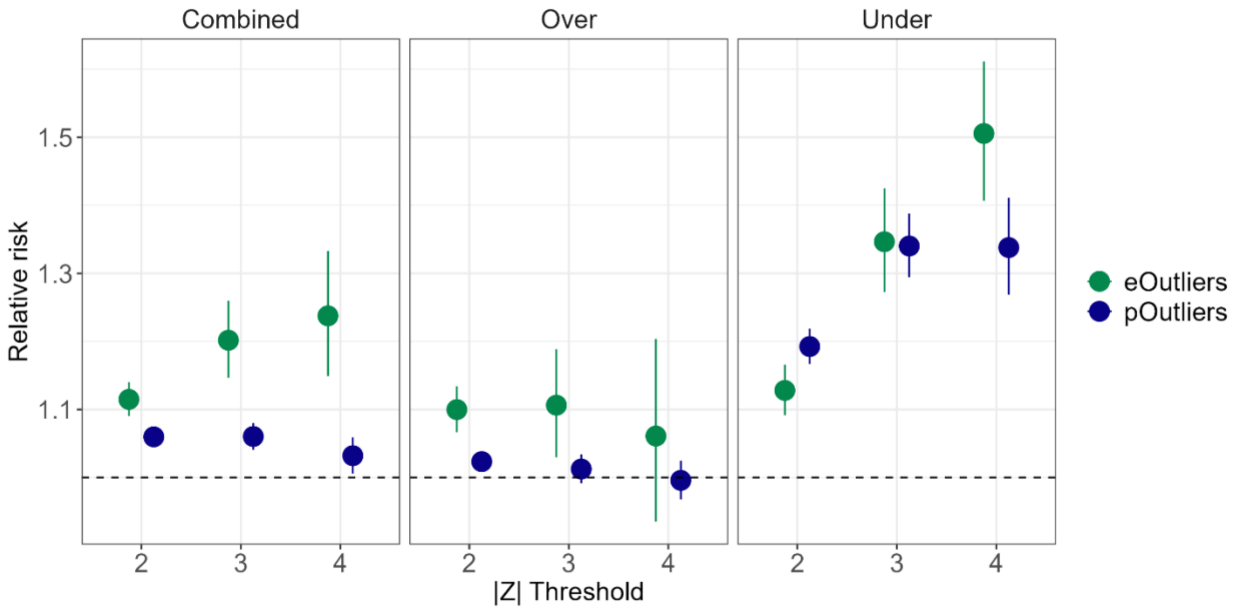

Figure S6. Enrichment of eOutliers restricted to the set of assayed protein genes, related to Figure 3. Estimates of relative risk of carrying a nearby rare variant (gene body +/- 10kb) across thresholds (x-axis) for eOutliers (green) identified across both exams for the subset of genes with protein measurements, and all pOutliers plotted for comparison (blue).

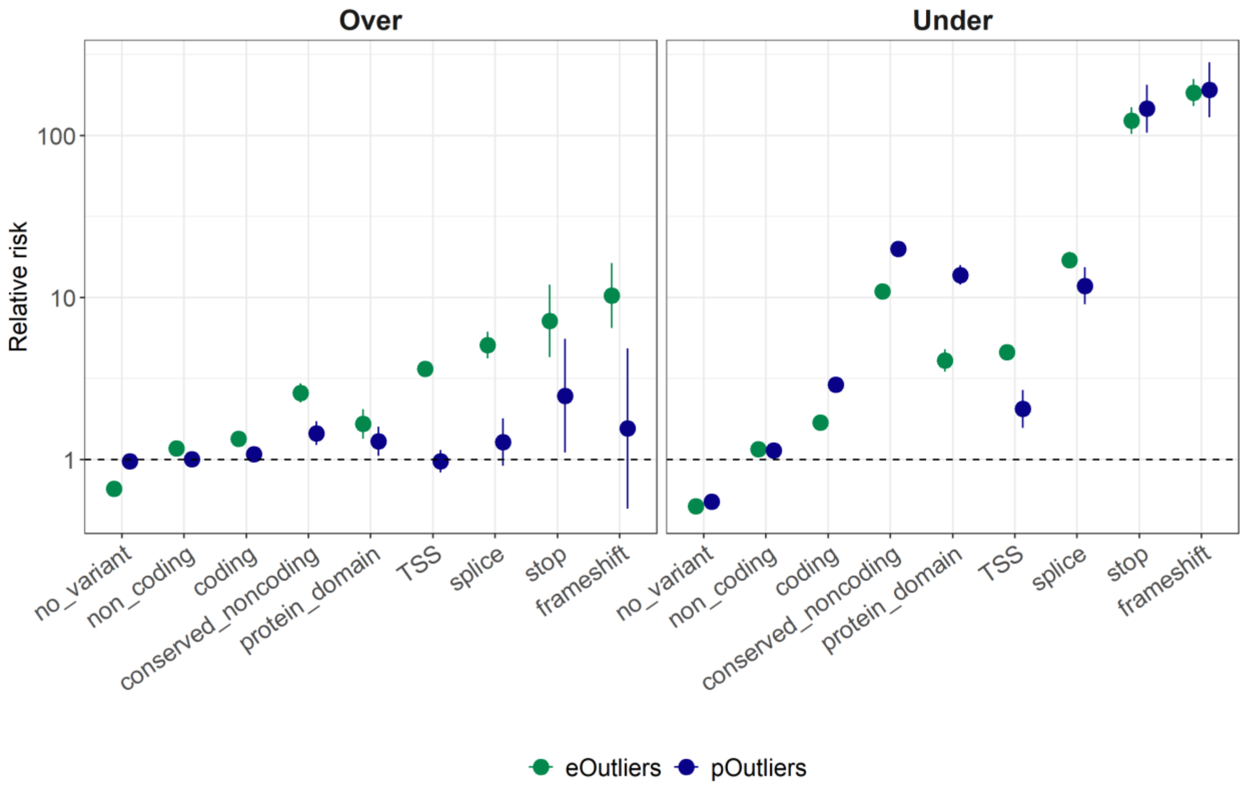

Figure S7. Enrichment of annotated rare variants nearby eOutliers and pOutliers, related to Figure 3. Estimates of relative risk of carrying a nearby rare variant (gene body +/- 10kb) for different types of variants (x-axis) for eOutliers (green) and pOutliers (blue) split by the direction of the effect.

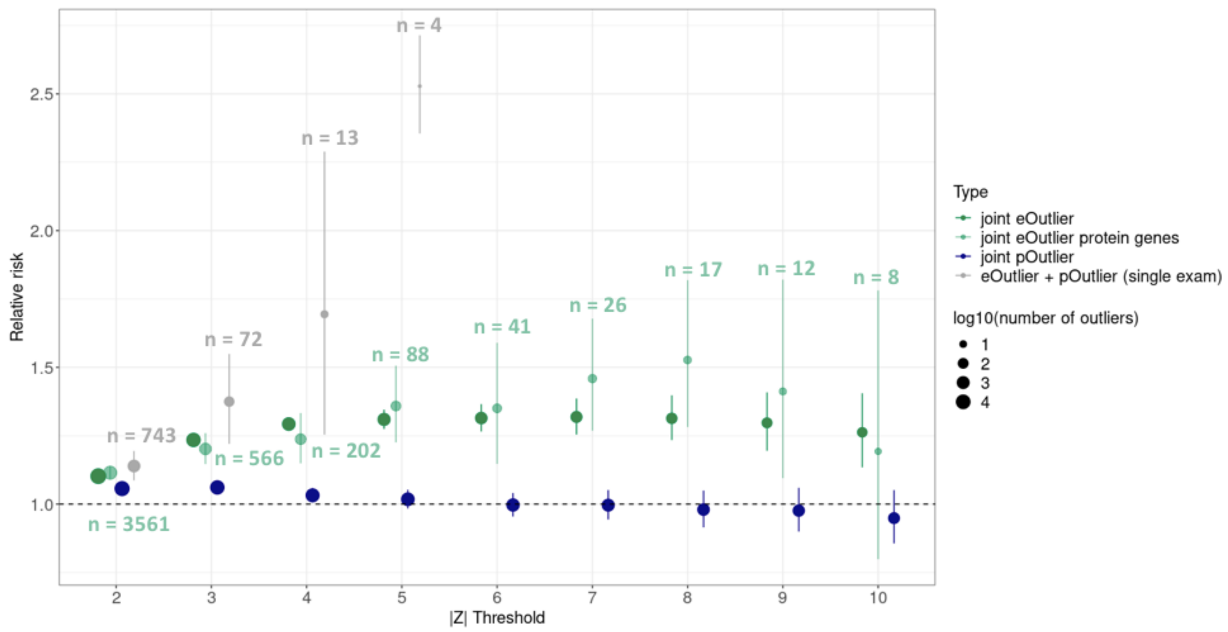

Figure S8. Enrichment of single time point overlapping outliers vs joint outliers in a single data type, related to Figure 3. (A) The relative risk of carrying a nearby rare variant (gene body +/- 10kb) across thresholds (x-axis) for eOutliers identified across both exams (green), the subset of joint eOutliers for genes also measured in protein (light green), pOutliers identified across both exams (blue), and overlapping eOutliers and pOutliers identified in a single exam (grey). The number of identified outliers are listed at each threshold for the filtered joint eOutlier set (light green) and the overlapping outlier set (grey), with the size of each point also varying by the log10(number of outliers) seen for that particular set and threshold combination.

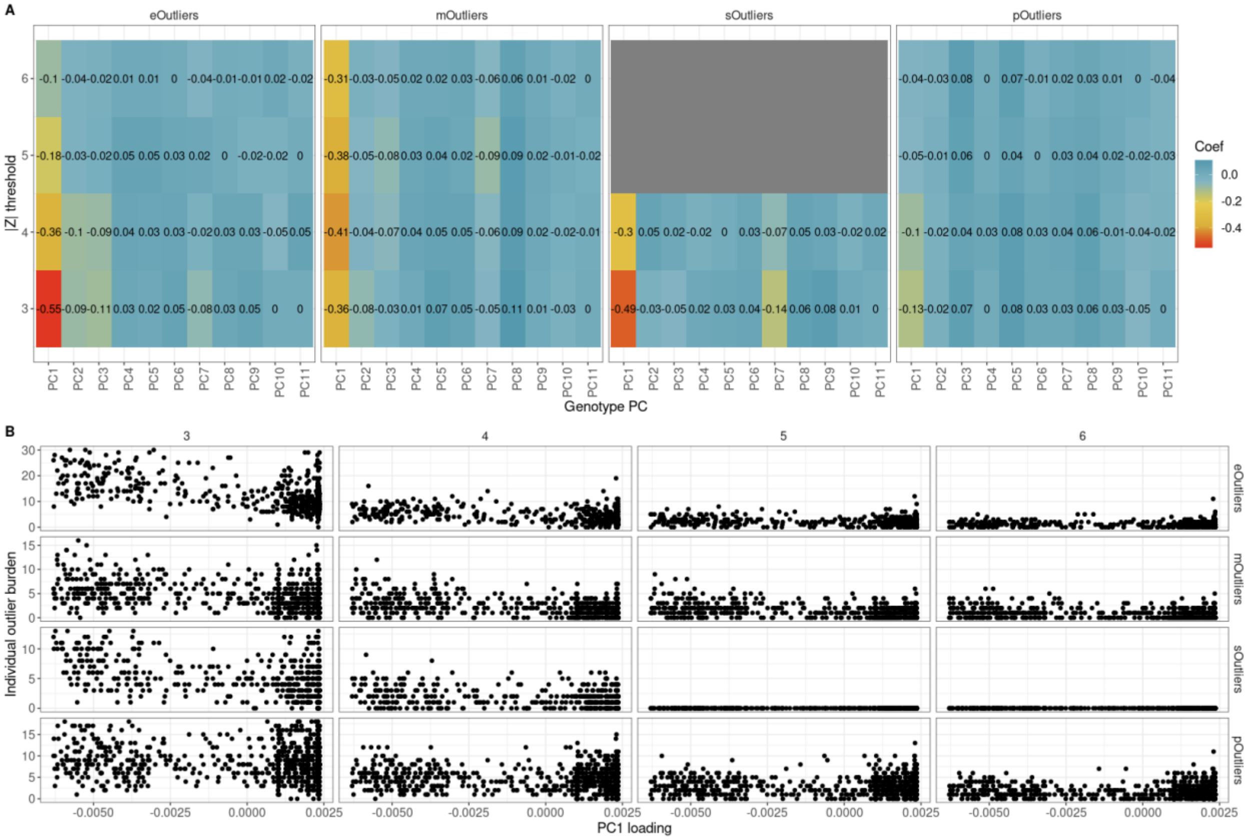

Figure S9. Correlation of individual outlier burden and genotype PCs, related to Figure 3. **(A)** The Pearson correlation of individual gene-level joint outlier burden across thresholds (y-axis) and individual genotype PC values (x-axis) for each gene-level outlier type. **(B)** Genotype PC1 values (x-axis) and individual outlier burden (x-axis) across thresholds (|Z| > 3, 4, 5, 6 in both exams) for each gene-level outlier type.

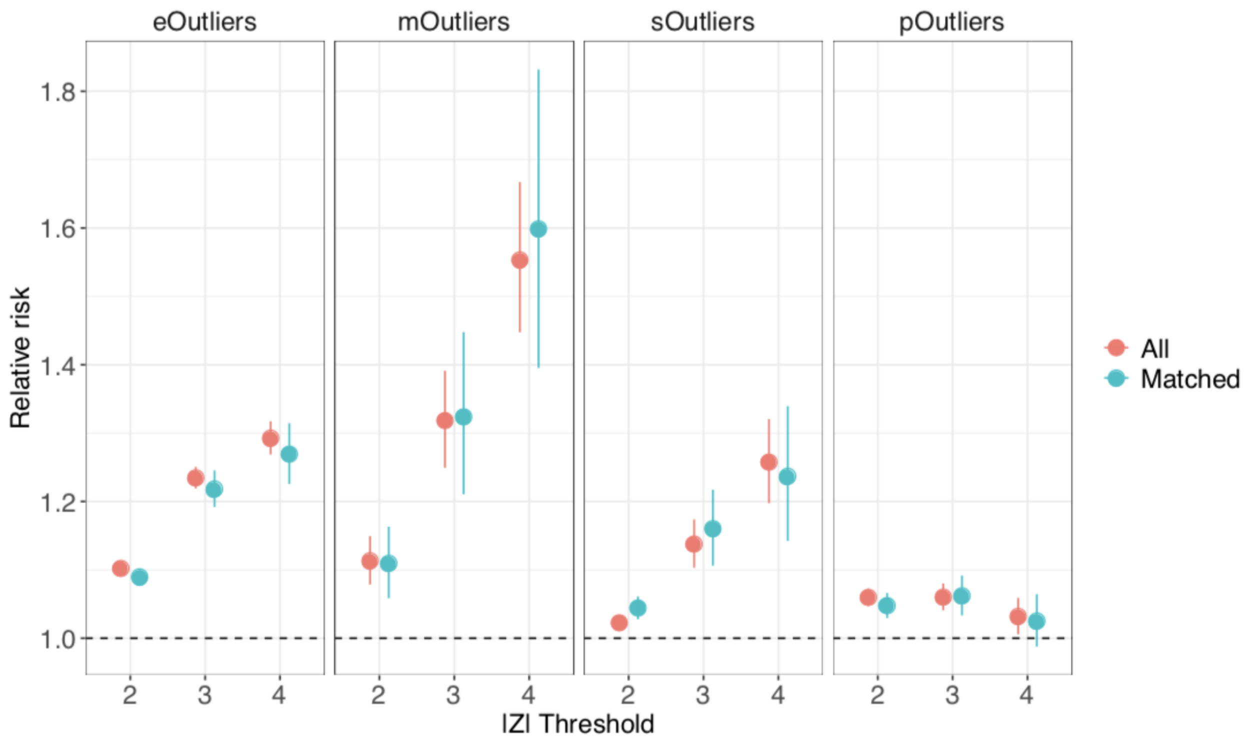

Figure S10. Enrichment of rare variants nearby gene-level outliers relative to all controls or a subset matched by genotype PCs, related to Figure 3. The relative risk of carrying a nearby rare variant (gene body +/- 10kb) across thresholds (x-axis) for gene-level joint outliers, considering as non-outliers all individuals with |Z| < 1 in both exams (pink) or selecting one non-outlier individual per outlier from all those with |Z| < 1 in both exams based on euclidean distance calculated across the top 11 genotype PC values (teal).

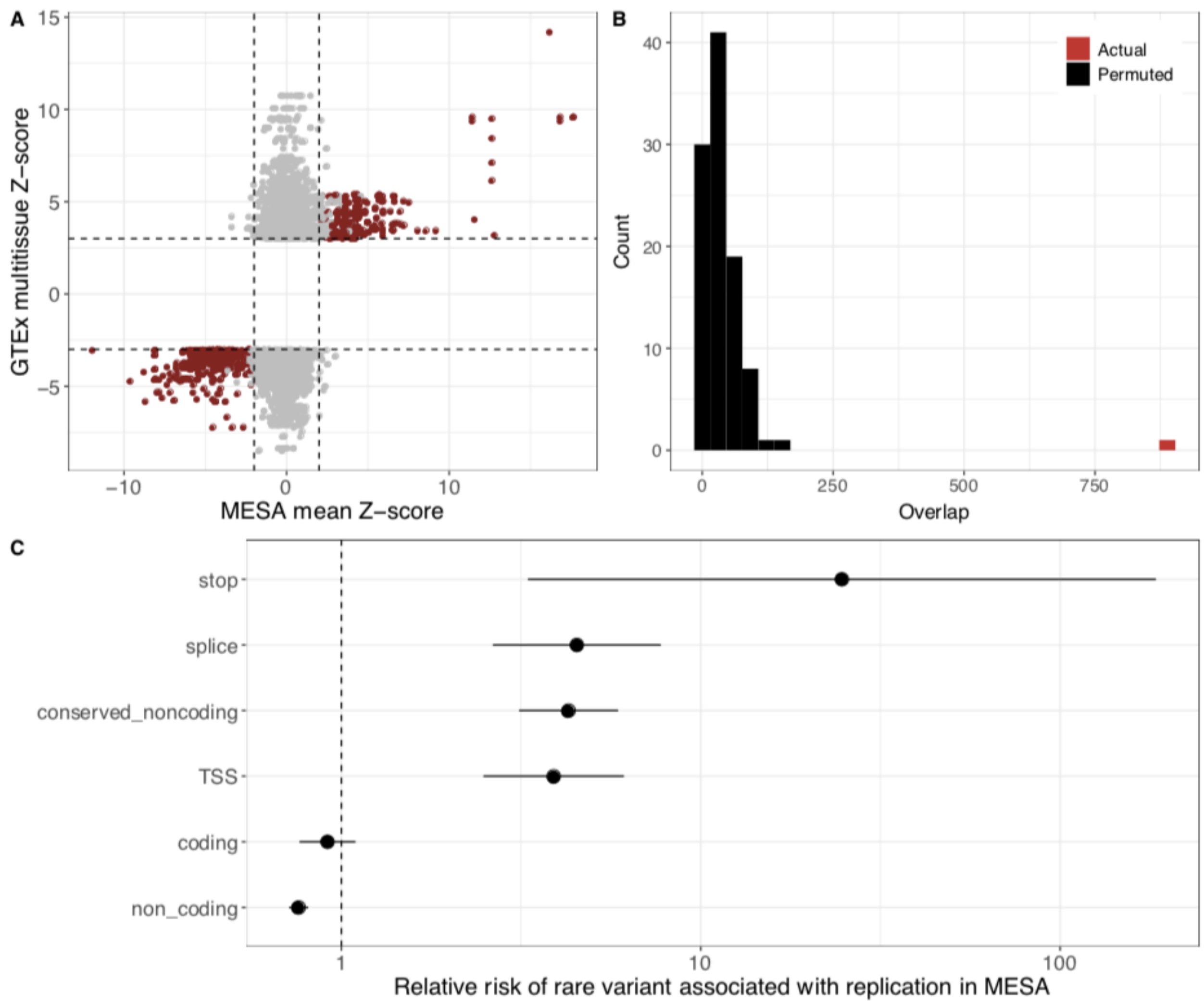

Figure S11. Replication of GTEx multitissue-eOutlier associated rare variants in MESA, related to Figure 3. (A) The mean expression Z-score across exams in MESA (x-axis) vs the median expression Z-score across tissues in GTEx (y-axis) for the set of variant-gene-individual instances where the same rare variant is carried by a GTEx and MESA individual and associated with multitissue outlier expression in GTEx. The dashed lines indicate the GTEx outlier threshold (|median Z| > 3) and the replication threshold in MESA (|Z| > 2 in both exams). Dark red indicates the instances that do replicate in both exams in MESA. **(B)** The number of instances in which the same rare variant is associated with outlier expression in GTEx and MESA (red), using a threshold of |Z| > 2 in both exams, vs the number observed after permuting expression Z-scores across all individuals in MESA, maintaining exam pairs. **(C)** The relative risk (x-axis) of a rare variant associated with outlier expression in GTEx replicating in MESA given that variant's annotation (y-axis). If a variant type was not associated with any replicating effect in MESA, it is not included here.

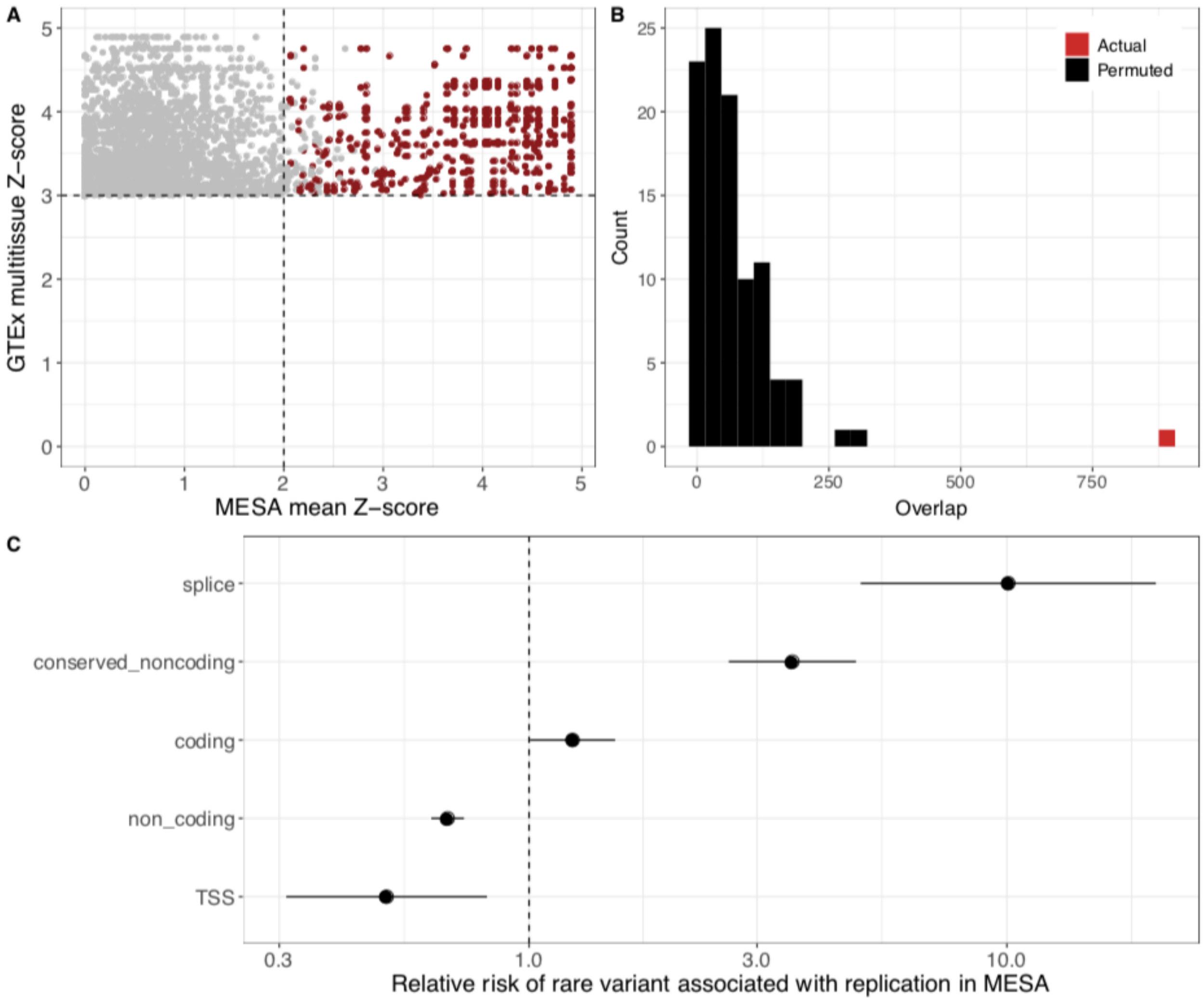

Figure S12. Replication of GTEx multitissue-sOutlier associated rare variants in MESA, related to Figure 3. (A) The mean splicing Z-score across exams in MESA (x-axis) vs the median splicing Z-score across tissues in GTEx (y-axis) for the set of variant-gene-individual instances where the same rare variant is carried by a GTEx and MESA individual and associated with multitissue outlier splicing in GTEx. The dashed lines indicate the GTEx outlier threshold (median Z > 3 or equivalently, median splicing p-value < 0.0027) and the replication threshold in MESA (Z > 2 in both exams). Dark red indicates the instances that do replicate in both exams in MESA. **(B)** The number of instances in which the same rare variant is associated with outlier splicing in GTEx and MESA (red), using a threshold of Z > 2 in both exams, vs the number observed after permuting splicing Z-scores across all individuals in MESA, maintaining exam pairs. **(C)** The relative risk (x-axis) of a rare variant associated with outlier splicing in GTEx replicating in MESA given that variant's annotation (y-axis). If a variant type was not associated with any replicating effect in MESA, it is not included here.

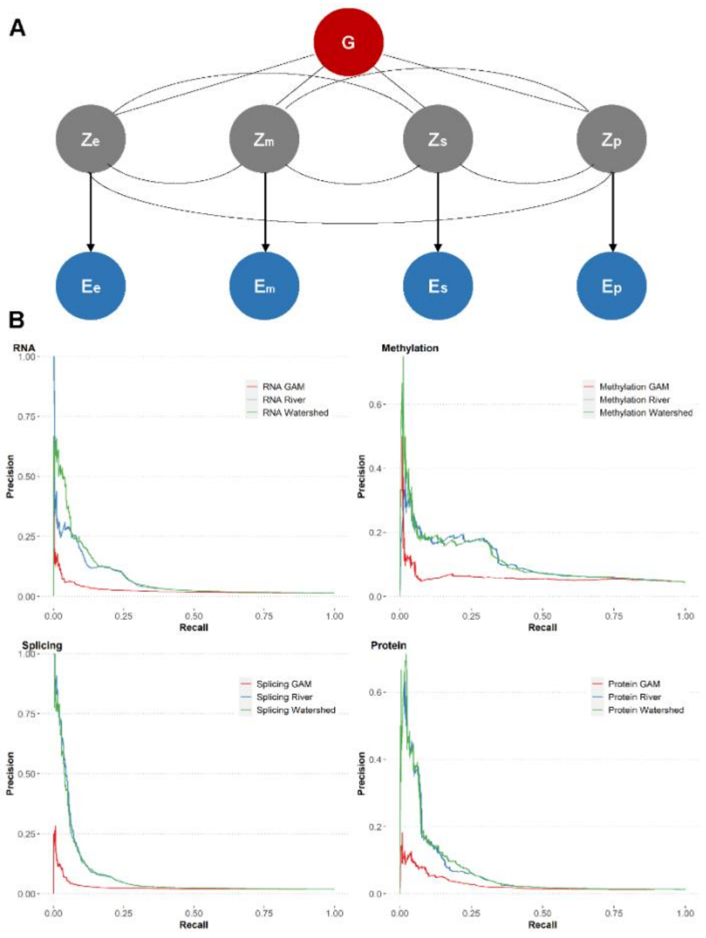

Figure S13. Multi-omic Watershed model, related to Figure 4. (A) Schematics of the Watershed hierarchical Bayesian model which is trained on (gene, individual) pairs consisting of genomic annotations aggregated across all rare variants (G), categorical variable E which represents observed outlier status of the gene in each signal (e – mRNA expression; m – methylation; s – splicing; and p – protein expression), and binary latent variables Z representing unobserved regulatory status on each signal. The Z layer is a fully connected layer which affords flexibility to model relationships across the regulatory cascade. **(B)** Precision-recall curves of Watershed models (green), River models (blue), and genomic annotation models (GAM, red) for each omic signal, evaluated against (gene, individual) pairs with the same set of rare variants nearby.

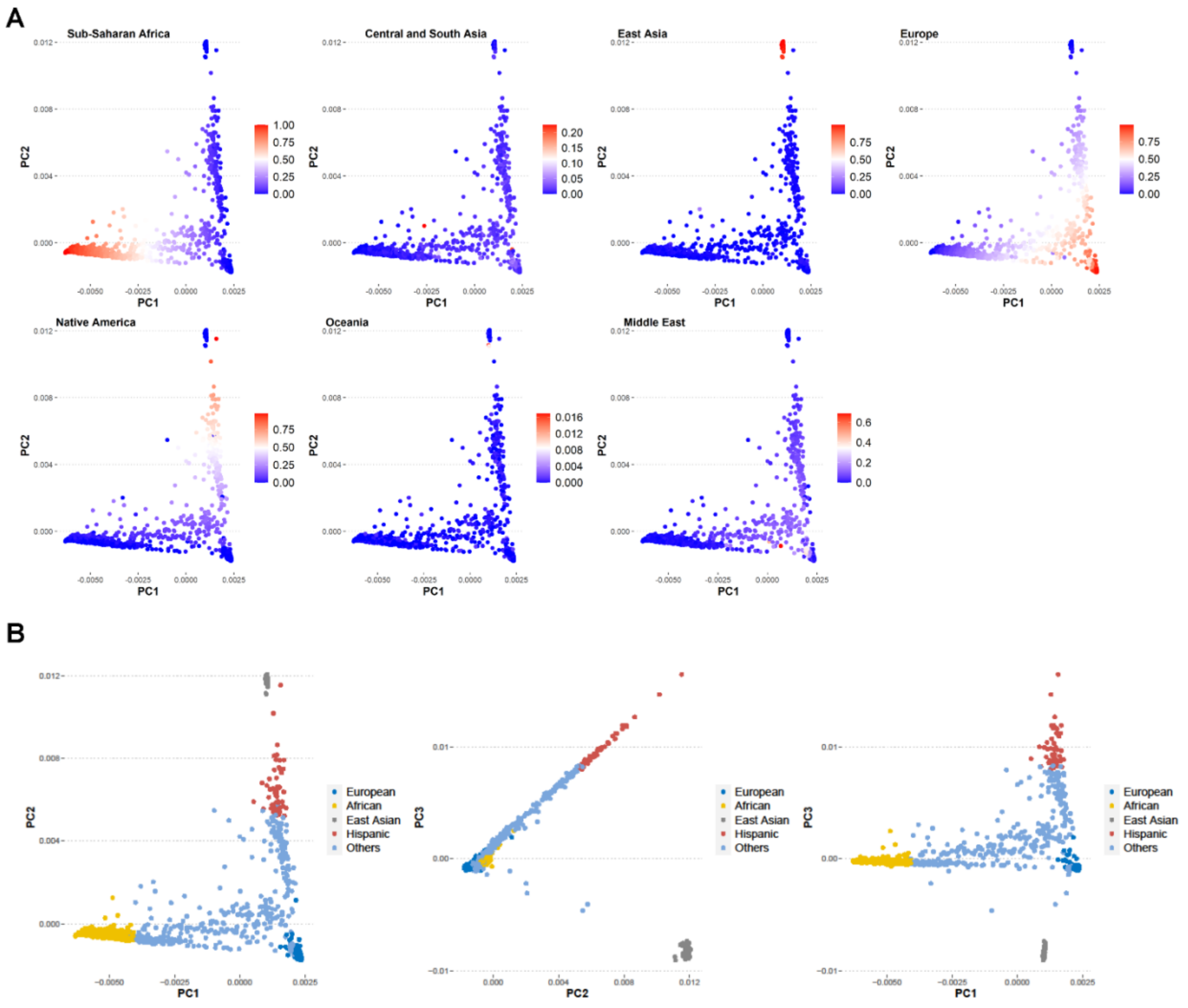

Figure S14. Population diversity in MESA, related to Figure 4. (A) Genotype principal component (PC) plots for N = 1319 individuals in MESA with multi-omic measurements, colored by inferred ancestry using RFMix (Maples et.al. 2013) based on the Human Genome Diversity Panel (HGDP) with seven super populations. **(B)** Genotype PC plots for the same set of individuals colored by population grouping obtained by setting thresholds on inferred ancestry in (A). The resulting populations have N = 426 Europeans, N = 270 Africans, N = 107 East Asians, and N = 54 Hispanic individuals.

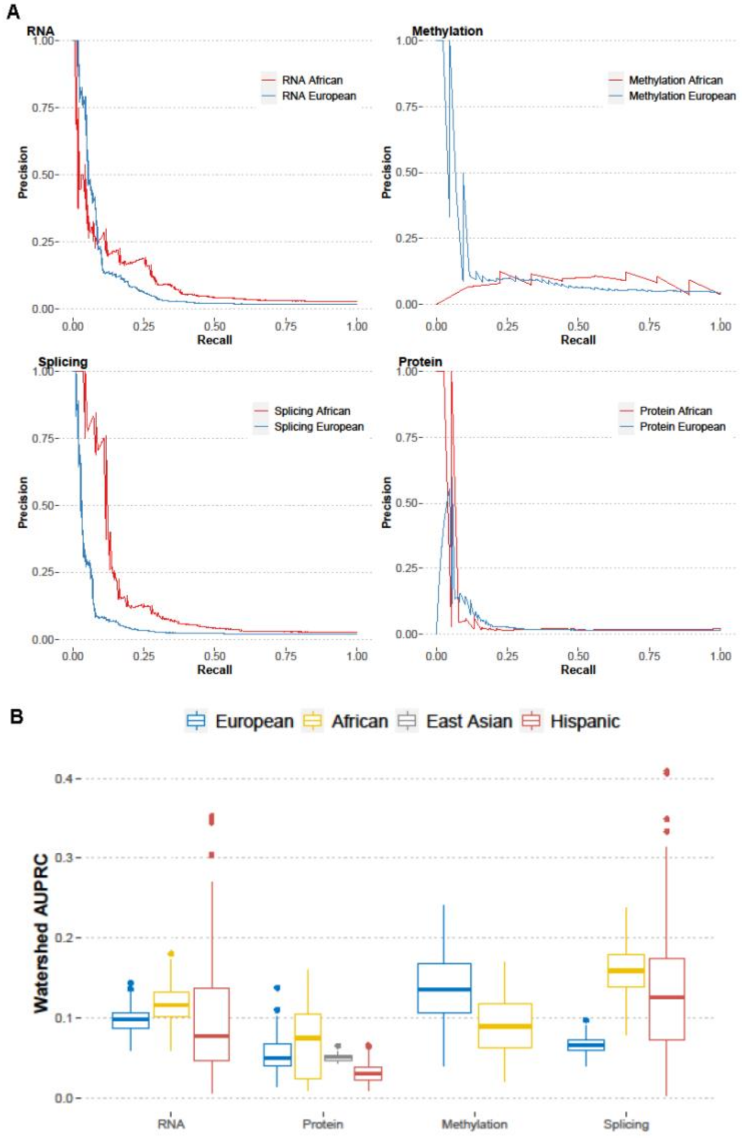

Figure S15. **Cross-population Watershed performance, related to Figure 4**. **(A)** Precision-recall curves for Watershed models trained on European individuals and evaluated on either European N2 pairs (blue) or African N2 pairs (red) across four omic signals. **(B)** Summary of 100 bootstrapped area under the precision-recall curves (AUPRC) for Watershed models trained on European individuals and evaluated on other populations in MESA across our omic signals. Shown are median and interquartile ranges. Only populations and signals with at least ten pairs of N2 individuals for evaluation are included.

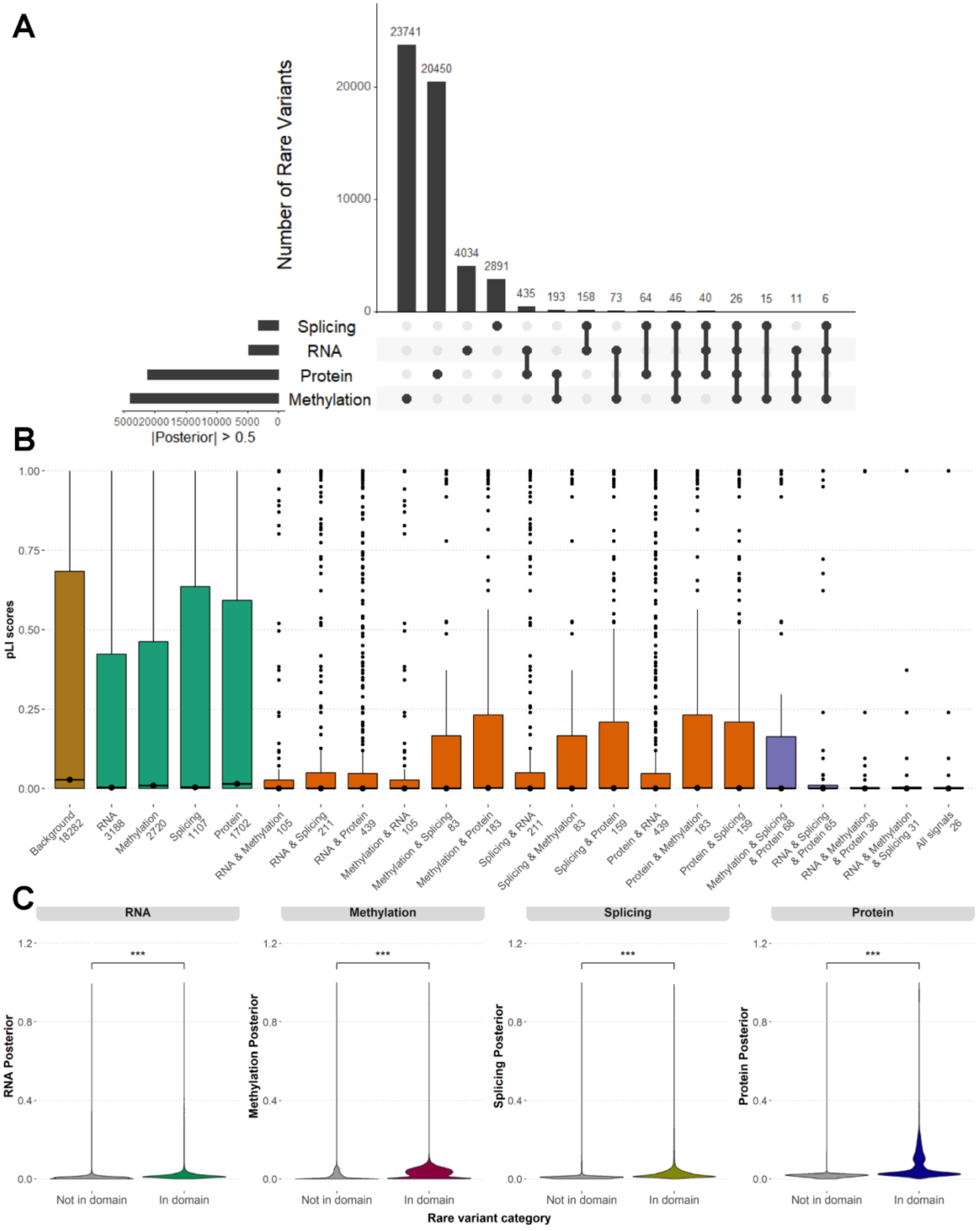

Figure S16. Cross-modality comparison of Watershed posteriors, related to Figure 5. (A) Upset plot of all rare variants passing 0.5 posterior threshold in each omic signal. (B)

Distribution of probability of loss of function intolerance (pLI) for genes with rare variants passing 0.5 posterior threshold in each signal individually (green), and combinations of two (orange), three (purple), and all four (red) signals. Background is represented by all genes with any omic measurement in MESA. Number of rare variants in each group is shown in the x-axis labels. Median and interquartile range is shown for each distribution. (C) Violin plots of Watershed posteriors in each signal for rare variants identified in protein domains, compared with those not in domains. *** $p < 0.001$, one-sided Wilcoxon rank-sum test on absolute value of posteriors between the two variant category.

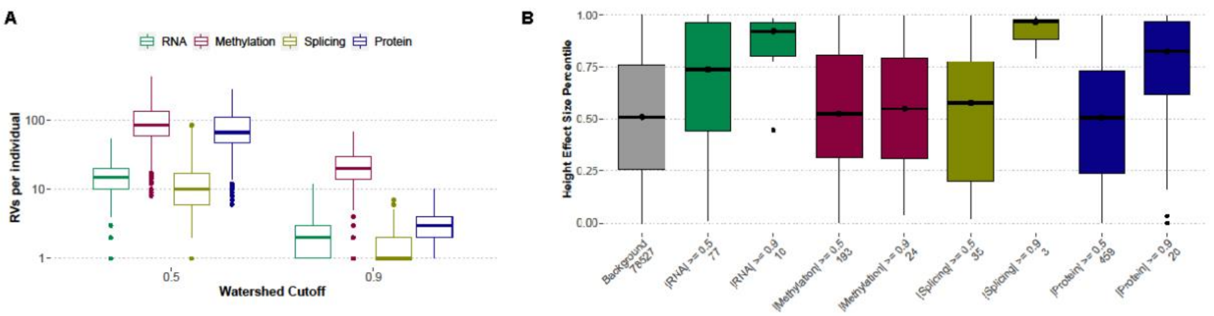

Figure S17. Assessment of Watershed performance for genes directly measured in each omic signal, related to Figure 5. (A) Number of rare variants per individual as prioritized by each omic signal at two levels of Watershed posterior cutoff 0.5 and 0.9. Individuals with a significantly large number of outlier expressions ("global outliers") are excluded. Similar to Figure 5A, except only genes which are directly measured in each omic signal are included. (B) Distribution of percentile normalized effect size for height (median and interquartile range) of all rare variants (background, gray), and those variants prioritized by Watershed in each signal. Only those rare variants mapped to genes with evidence of causing abnormal body height which are directly measured in each omic signal are included.

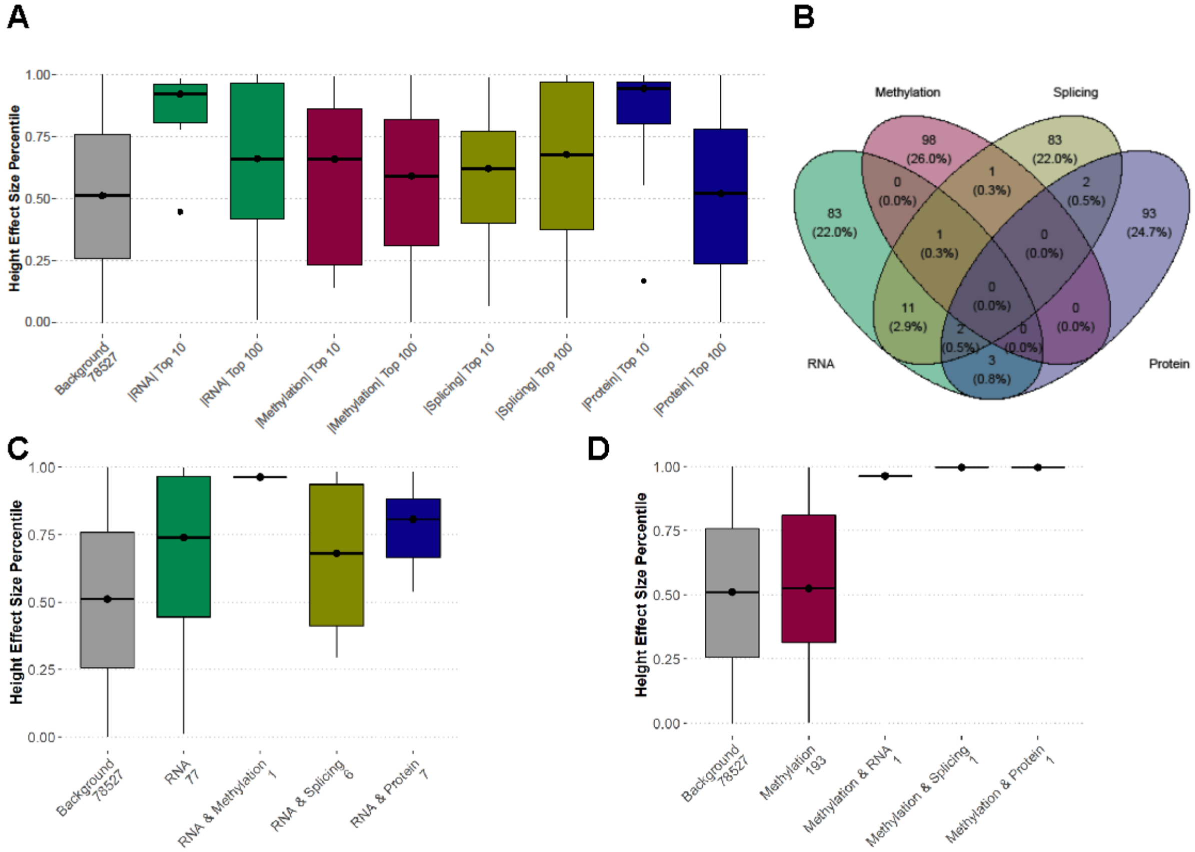

Figure S18. Evaluation of the effect size for height for rare variants prioritized by the multi-omic Watershed model, related to Figure 5. (A) Distribution of percentile normalized effect size for height (median and interquartile range) of all rare variants (background, gray), and those rare variants prioritized by multi-omic Watershed in each signal with top 10 or top 100 highest posteriors. (B) Venn diagram of top 100 rare variants with highest posteriors across signals. (C – D) Distribution of percentile normalized effect size for height (median and interquartile range) for rare variants prioritized by a single signal at a posterior threshold of 0.5 (C – RNA; D – methylation) and combined with another signal.

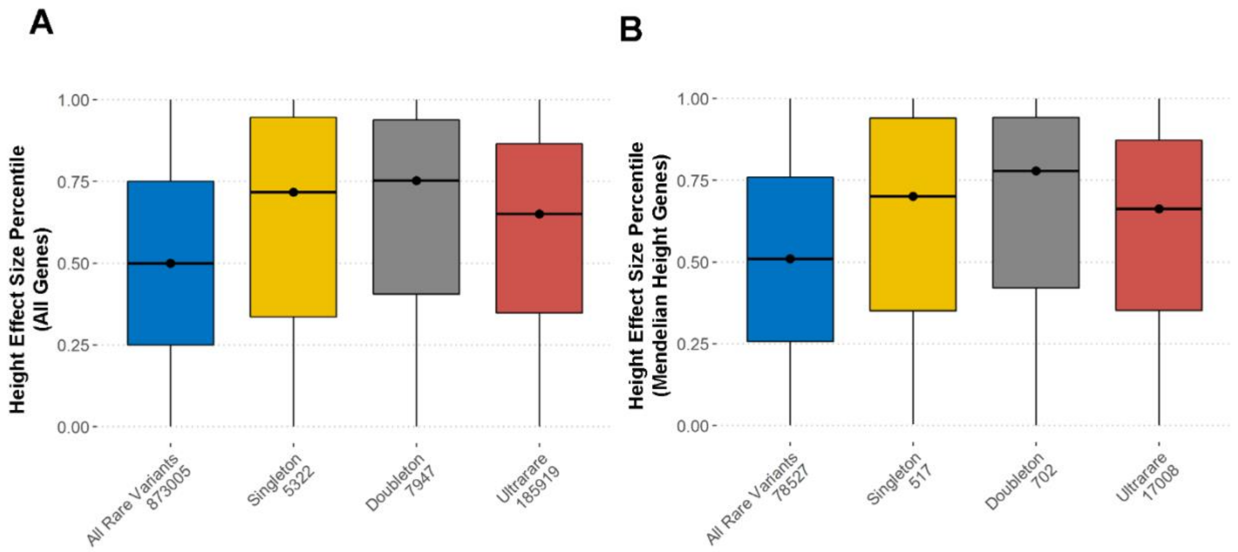

Figure S19. Evaluation of the effect size for height by minor allele frequency (MAF), related to Figure 5. Distribution of percentile normalized effect size for height (median and interquartile range) for all rare variants (blue), singletons (yellow), doubletons (grey), and ultrarare variants (MAF < 0.1%) across all genes **(A)** or N = 1,314 genes with evidence causing abnormal body height (Mendelian height genes, **B**).

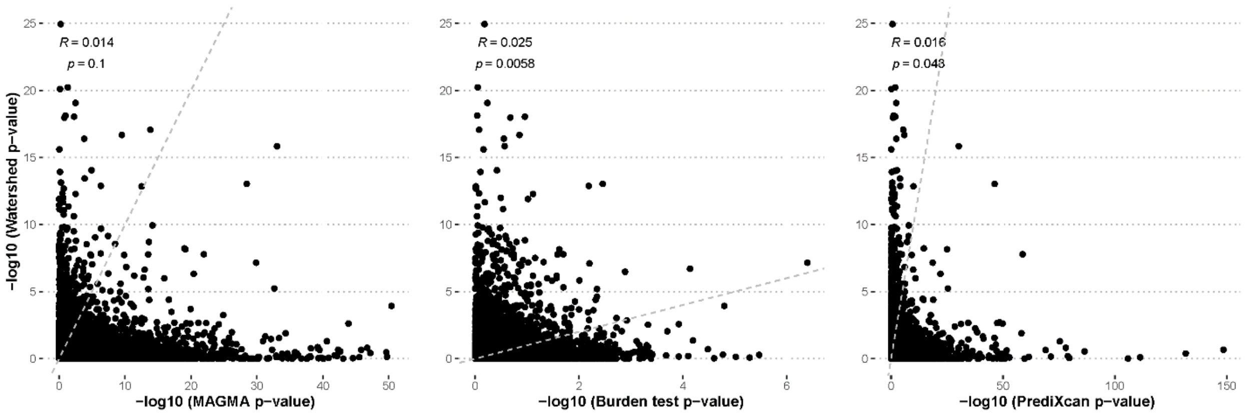

Figure S20. Comparison of collapsing analysis based on Watershed posteriors with other gene prioritization methods, related to Figure 5. Scatter plots of -log10 p-values of gene tests using Watershed posteriors as weights (y-axis) against those obtained from MAGMA **(A)**, burden test **(B)**, and PrediXcan **(C)** on height. Dotted lines represent the diagonal.

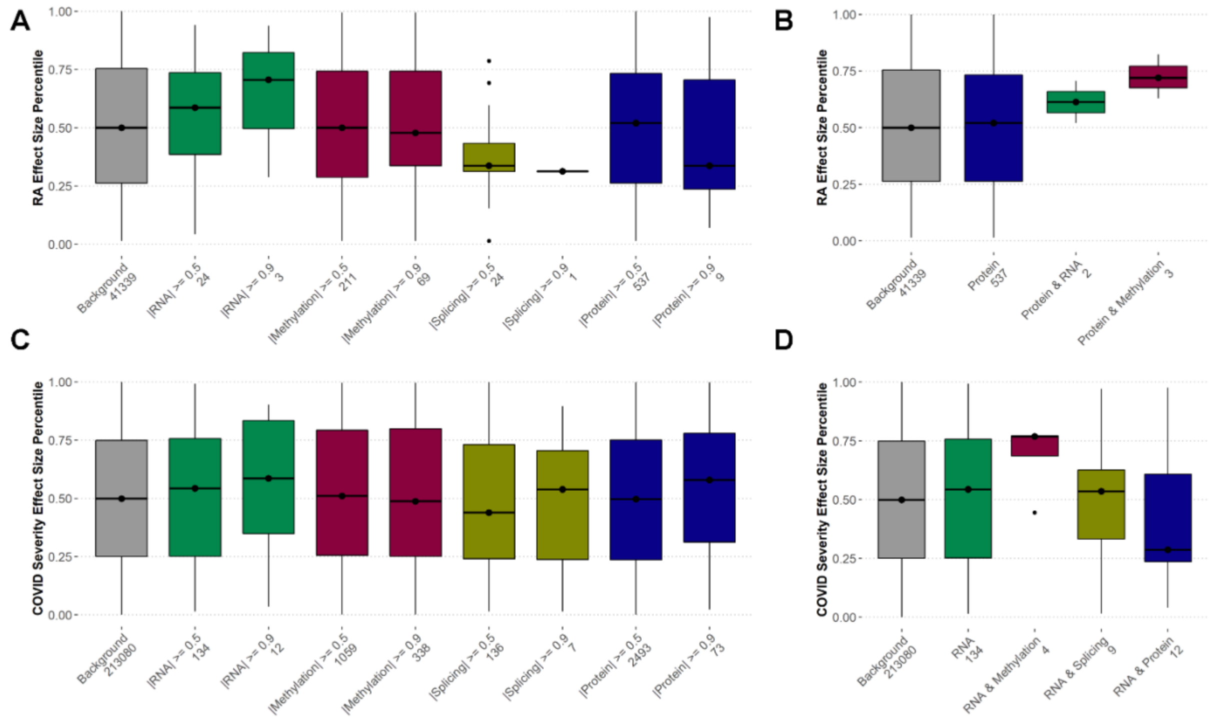

Figure S21. Evaluation of Watershed prioritized rare variants in two immune diseases, related to Figure 5. (A) Distribution of percentile normalized effect size for rheumatoid arthritis (RA, median and interquartile range) for all rare variants (background, grey), and those rare variants prioritized by multi-omic Watershed in each signal at two posterior threshold values. Only rare variants mapped to genes with evidence of association with RA as reported by Open Targets are shown (N = 4,769 genes). **(B)** RA effect size distribution as in **(A)** but with rare variants prioritized by protein (blue) or protein combined with another signal at a posterior threshold of 0.5. **(C)** Distribution of percentile normalized effect size for COVID-19 severity for all rare variants, and those rare variants prioritized by multi-omic Watershed in each signal at two posterior threshold values. Only rare variants mapped to genes with evidence of association with COVID-19 as reported by Open Targets are shown (N = 2,232 genes). **(D)** COVID-19 severity effect size distribution as in **(C)** but with rare variants prioritized by RNA (green) or RNA combined with another signal at a posterior threshold of 0.5.

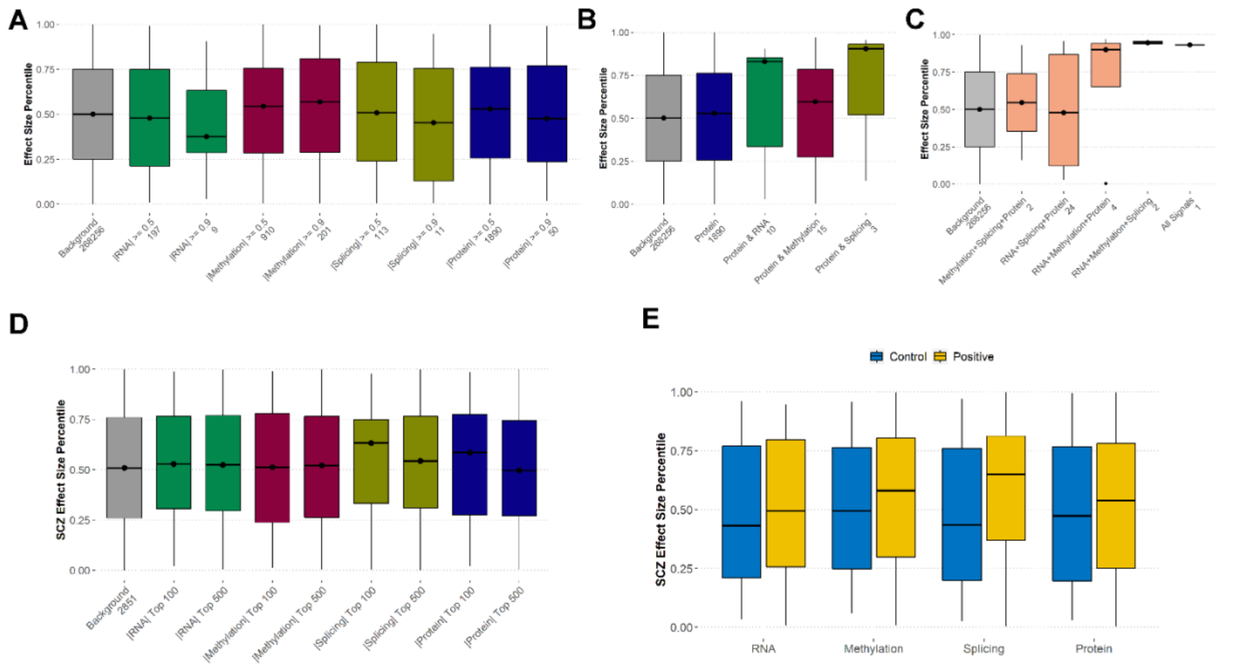

Figure S22. Evaluation of Watershed prioritized rare variants in two neurological diseases, related to Figure 5. (A) Distribution of percentile normalized effect size for Alzheimer's Disease (AD, median and interquartile range) for all rare variants (background, grey), and those rare variants prioritized by multi-omic Watershed in each signal at two posterior threshold values. Only rare variants mapped to genes with evidence of association with AD as reported by Open Targets are shown (N = 7,103 genes). **(B)** AD effect size distribution as in **(A)** but with rare variants prioritized by protein (blue) or protein combined with another signal at a posterior threshold of 0.5. **(C)** AD effect size distribution as in **(A)** but with rare variants prioritized by three or four omic signals. **(D)** Distribution of percentile normalized effect size for schizophrenia (SCZ, median and interquartile range) of all rare variants (background, gray), and those rare variants prioritized by multi-omic Watershed in each signal with top 100 or top 500 highest posteriors. Only rare variants mapped to genes with evidence of association with AD as reported by Open Targets are shown (N = 5,463 genes). **(E)** Distribution of SCZ effect size of rare variants mapped to MAGMA prioritized genes ("Positive", MAGMA z > 2, N = 5,378 genes, yellow) and control genes ("Control", MAGMA z < 0, N = 4,092 genes, blue) at a posterior threshold of 0.2 in each omic signal.

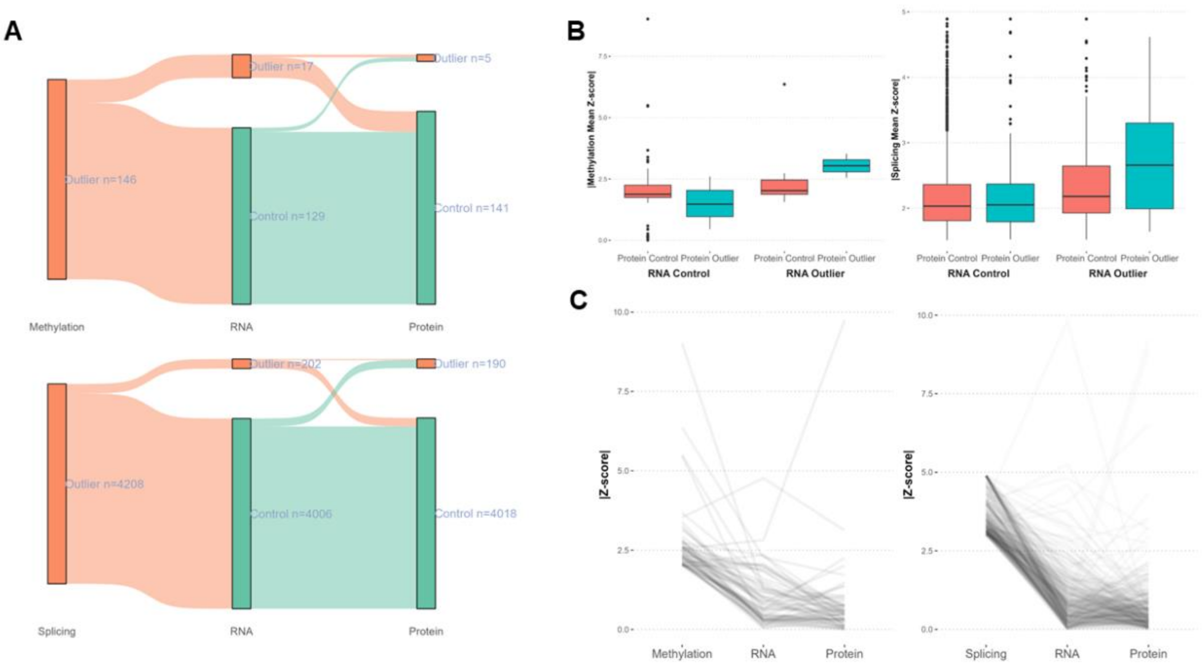

Figure S23. Propagation of outlier signals across the regulatory cascade, related to Figure 5. (A) Sankey plots showing the number of outliers ([gene, individual] pairs) in methylation (top) and splicing (bottom) which survive to become outliers in RNA and protein signals. **(B)** Distribution of mean Z-scores across two visits for outliers in methylation (left) and splicing (right), stratified by the outlier status in later omic signals (RNA and protein). **(C)** Trend lines showing changes in Z-scores for methylation (left) and splicing (right) outliers as they propagate to RNA and protein signals. All outliers were defined at Z-score threshold of 2, with the exception of splicing outliers in (**C)** which showed outliers at Z > 3 for visual clarity.
